# The m^6^A modification mediated-lncRNA POU6F2-AS1 reprograms fatty acid metabolism and facilitates the growth of colorectal cancer via upregulation of FASN

**DOI:** 10.1186/s12943-024-01962-8

**Published:** 2024-03-16

**Authors:** Tao Jiang, Junwen Qi, Zhenyu Xue, Bowen Liu, Jianquan Liu, Qihang Hu, Yuqiu Li, Jing Ren, Hu Song, Yixin Xu, Teng Xu, Ruizhi Fan, Jun Song

**Affiliations:** 1 Department of General Surgery, The Affiliated Hospital of Xuzhou Medical University, Xuzhou, Jiangsu 221006 China; 2 Institute of Digestive Diseases, Xuzhou Medical University, Xuzhou, Jiangsu 221002 China; 3 Affiliated First Clinical College, Xuzhou Medical University, Xuzhou, Jiangsu 221004 China; 4 Central Laboratory, The Affiliated Hospital of Xuzhou Medical University, Xuzhou, Jiangsu 221002 China; 5 Jiangsu Key Laboratory of Brain Disease Bioinformation, Research Center for Biochemistry and Molecular Biology, Xuzhou Medical University, Xuzhou, Jiangsu 221004 China; 6 Department of Radiation Oncology, The Affiliated Hospital of Jiangsu University, Zhenjiang, Jiangsu 212001 China

**Keywords:** Colorectal cancer, POU6F2-AS1, Fatty acid metabolism, Growth

## Abstract

**Background:**

Long noncoding RNAs (lncRNAs) have emerged as key players in tumorigenesis and tumour progression. However, the biological functions and potential mechanisms of lncRNAs in colorectal cancer (CRC) are unclear.

**Methods:**

The novel lncRNA POU6F2-AS1 was identified through bioinformatics analysis, and its expression in CRC patients was verified via qRT–PCR and FISH. In vitro and in vivo experiments, such as BODIPY staining, Oil Red O staining, triglyceride (TAG) assays, and liquid chromatography mass spectrometry (LC-MS) were subsequently performed with CRC specimens and cells to determine the clinical significance, and functional roles of POU6F2-AS1. Biotinylated RNA pull-down, RIP, Me-RIP, ChIP, and patient-derived organoid (PDO) culture assays were performed to confirm the underlying mechanism of POU6F2-AS1.

**Results:**

The lncRNA POU6F2-AS1 is markedly upregulated in CRC and associated with adverse clinicopathological features and poor overall survival in CRC patients. Functionally, POU6F2-AS1 promotes the growth and lipogenesis of CRC cells both in vitro and in vivo. Mechanistically, METTL3-induced m^6^A modification is involved in the upregulation of POU6F2-AS1. Furthermore, upregulated POU6F2-AS1 could tether YBX1 to the FASN promoter to induce transcriptional activation, thus facilitating the growth and lipogenesis of CRC cells.

**Conclusions:**

Our data revealed that the upregulation of POU6F2-AS1 plays a critical role in CRC fatty acid metabolism and might provide a novel promising biomarker and therapeutic target for CRC.

**Supplementary Information:**

The online version contains supplementary material available at 10.1186/s12943-024-01962-8.

## Background

Colorectal cancer (CRC) is the most common malignancy of the digestive system, and the second leading cause of cancer-related mortality worldwide [1]. Although significant progress has been made in surgical resection, chemotherapy, targeted therapy and immunotherapy for CRC, the prognosis of CRC patient is still unfavourable [2–4]. CRC hallmarks include rapid proliferation, which requires nutrients and facilitates the removal of cellular waste in nutrient-poor environments [5]. Research progress in the last decade has demonstrated that metabolic reprogramming is a pivotal general characteristic of cancer, that allow CRC cells to produce energy sufficient for rapid proliferation [6]. Accumulating evidence has confirmed aberrant metabolic reprogramming as a critical contributor to CRC initiation, proliferation and metastasis [7]. Hence, elucidating the regulatory mechanisms linking colorectal carcinogenesis factors and metabolic enzymes may provide promising avenues for improving CRC prognosis and developing effective therapeutic strategies.

As a critical metabolic feature of cancer cells, lipid metabolism has been widely reported to participate in cancer initiation, progression and drug resistance [8]. Fatty acid synthase (FASN) is a central enzyme of lipid metabolism that can catalyse de novo fatty acid (FA) biosynthesis to promote cell growth and survival [9]. For instance, loss of FBXW7β function facilitates FASN protein stability and promotes lipogenesis and growth in CRC [10]. However, the mechanisms underlying the dysregulation of lipogenesis and aberrant FASN expression in CRC still need to be clarified.

Long noncoding RNAs (lncRNAs) are transcripts longer than 200 nucleotides in length that have limited or no ability to encode proteins, but they can regulate protein-coding gene expression by modulating mRNA transcription, translation, and post- translational modification [11, 12]. Over the past decade, lncRNAs have been reported to play vital roles in various cellular functions in CRC, including proliferation, apoptosis, migration and invasion [13]. Previously, we reported that lncRNAs played important roles in CRC tumorigenesis and progression [14–16]. Furthermore, several lncRNAs were reported to be critical players in lipid metabolism modulation by CRC cells. For example, ZFAS1 binds with PABP2 to stabilize SREBP1 mRNA, thus facilitating lipid accumulation in CRC cells [17]. SNHG16 acts as a “sponge” for miRNAs to promote lipid metabolism by upregulating SCD [18]. However, additional crosstalk between lncRNAs and metabolic enzymes or processes still needs to be characterized.

In the present study, we identified a novel lncRNA, POU6F2-AS1, whose expression is significantly upregulated in CRC; this upregulation phenotype is positively associated with poor clinical outcomes in CRC patients. Moreover, we found that POU6F2-AS1 drives the growth and lipogenesis of CRC cells in vitro and in vivo, and these effects partially dependent on YBX1-mediated transcriptional activation of FASN. We also demonstrated that METTL3-induced m^6^A modification maintains POU6F2-AS1 stability and increases its expression. Taken together, these findings revealed that POU6F2-AS1 may serve as a promising biomarker and therapeutic target for CRC.

## Materials and methods

### Clinical specimens

This study was reviewed and approved by the Ethics Committee of the Affiliated Hospital of Xuzhou Medical University (XYFY2022-KL473-01), and consent was obtained from all participants. A cohort comprising 84 pairs of CRC tissues and corresponding adjacent normal tissues (ANTs) was collected from CRC patients who underwent radical resection or palliative resection at the Department of Gastrointestinal Surgery of the Affiliated Hospital of Xuzhou Medical University between January 2023 and April 2023 and was used for extracting RNA to detect the expression level of POU6F2-AS1. In addition, a cohort of 60 pairs consisting of paraffin-embedded CRC tissues and corresponding ANTs was collected for tissue microarray (TMA) analysis. All CRC patients had a pathologically confirmed diagnosis and did not receive preoperative radiotherapy or chemotherapy. Clinicopathological characteristics, such as patient age and sex and tumour size and depth of invasion, etc., were also obtained.

### Cell lines and culture

The human normal colorectal epithelial cell line FHC was obtained from the American Type Culture Collection (Manassas, VA, USA), while several human CRC cell lines (HCT116, DLD1, LoVo, SW620, HT29 and SW480) were obtained from the Cell Bank of the Chinese Academy of Sciences (Shanghai, China). HCT116 cells were cultured in McCoy´s 5 A medium supplemented with 10% foetal bovine serum (FBS) (Gibco, MA, USA), while SW620 cells were cultured in L-15 medium (Gibco, MA, USA). DLD1, HT-29, and LoVo cells were cultured in RPMI 1640 medium (Gibco, MA, USA), and FHC and SW480 cells were cultured in DMEM/high-glucose medium (Gibco, MA, USA). All the cells were cultured in a constant temperature incubator at 37 °C containing 5% CO_2_ and subjected to mycoplasma testing to confirm that they were free of contamination before use.

### BODIPY staining

The lipophilic fluorescence dye BODIPY (Molecular Probes, MA, USA) was used to monitor the content of neutral lipids in CRC cells. The transfected CRC cells were inoculated into 24-well plates containing coverslips and fixed with 4% paraformaldehyde for 30 min after 24 h. The cells were subsequently incubated with 2 µM BODIPY working solution in the dark for 30 min at 37 °C. The staining procedure was stopped by washing the cells with PBS, after which the nuclei were counterstained with Hoechst 33,342 (Beyotime, Shanghai, China) for 10 min. Representative images were taken with a confocal laser scanning microscope (Carl Zeiss, Germany).

### Oil Red O staining

Oil Red O staining was performed using Oil Red O Saturated Solution (Solarbio Life Sciences, Beijing, China) according to the manufacturer’s instructions. Briefly, an Oil Red O working solution was prepared by mixing 3 parts of Oil Red O saturated solution with 2 parts deionized water. The transfected CRC cells were inoculated on 14 mm round coverslips in 24-well plates, washed twice with PBS after 24 h, and fixed in 4% paraformaldehyde for 30 min before staining. For frozen tissue sectioning, optimal cutting temperature (OCT)-embedded tissue was cut into 8 μm sections, which were fixed in 4% paraformaldehyde for 30 min before staining. The cells or tissue sections were incubated in 60% isopropanol for 30 s and then in Oil Red O working solution for 20 min at room temperature. Then sections were subsequently washed with 60% isopropanol and deionized water again, counterstained with haematoxylin (Solarbio Life Sciences, Beijing, China), and photographed by an Olympus microscope (Tokyo, Japan). The Oil Red O staining area was calculated as Oil Red O^+^ area divided by the area of hematoxylin staining and normalized to the average of the control.

### Triglyceride (TAG) assay

For the quantitative estimation of triglycerides in cells, a Triglyceride Assay Kit (Promega, WI, USA) was used in accordance with the manufacturer’s protocols. Transfected cells were inoculated into 96-well plates and subjected to a triglyceride assay after 24 h. The cells were washed twice with PBS, and glycerol detection reagent was added, followed by incubation at room temperature for 1 h to detect luminescence. The luminescence signal was measured using a Tecan Spark zymography (Zurich, CH).

### Biotinylated RNA pull-down assay

In brief, T7 promoter-containing DNA was obtained by PCR amplification and purified using the TIANgel Purification Kit (TIANGEN BIOTECH, Beijing, China). The purified T7 promoter-containing DNA was incubated with a biotin RNA labelling mixture (Promega Corporation, MA, USA), T7 RNA polymerase (Thermo Fisher Scientific, MA, USA) and an RNase inhibitor for 2 h at a constant temperature of 37 °C in a PCR instrument and purified using an RNAclean Kit (TIANGEN BIOTECH, Beijing, China) to obtain the biotin-labelled RNA probe. Biotinylated RNAs were incubated with protein extracted from CRC cells and mixed with precleared streptavidin agarose resin (Thermo Fisher Scientific, MA, USA). The RNA-protein complexes were obtained by collecting the agarose resin by centrifugation, and the proteins bound therein were eluted and denatured, followed by electrophoresis on SDS-PAGE gels. Afterwards, the proteins were silver stained, and the differential bands were excised for mass spectrometry analysis (Shanghai Applied Protein Technology Co. Ltd., China). To identify which specific region of POU6F2-AS1 interacts with YBX1, we truncated POU6F2-AS1.

### Luciferase reporter assay

The luciferase plasmid was constructed by Gene Create Biologicals (Wuhan, China). To investigate the transcriptional regulation of FASN, two different fragments of the FASN promoter region were PCR amplified and subsequently inserted into the KpnI/XhoI site upstream of firefly luciferase in the pGL3-Basic vector. Cells with or without knockdown or overexpression of YBX1/POU6F2-AS1 were transfected with the luciferase plasmid. Firefly luciferase activity was measured 48 h later using a Dual Luciferase Reporter Gene Assay Kit (Beyotime, Shanghai, China) according to the manufacturer’s instructions, with Renilla luciferase serving as a transfection control.

### Chromatin immunoprecipitation (ChIP) assay

The ChIP assay was performed using the BeyoChIP™ ChIP Assay Kit (Beyotime, Shanghai, China) according to the manufacturer’s instructions. Briefly, 1 × 10^7^ CRC cells were prepared and then cross-linked with 1% formaldehyde for 15 min at room temperature. The cross-linking process was quenched by adding 0.125 M glycine. Chromatin was isolated with the lysis buffer provided in the kit. Afterwards, sonication was performed to shear the DNA to 200–1000 bp. Immunoprecipitation of cross-linked protein/DNA was performed by incubating anti-YBX1 antibody (Proteintech, IL, USA) or normal rabbit IgG (Proteintech, IL, USA) with sheared chromatin overnight at 4 °C followed by incubation with Protein A/G Magnetic Beads for 2 h. Subsequently, cross-links of protein/DNA were released with 5 M NaCl, and the DNA was purified using a PCR Clean Up Kit (Beyotime, Shanghai, China). Finally, the purified DNA was subjected to qRT–PCR using the P1-P9 primer sequences listed in Additional file 1: Table S4.

### Patient-derived organoid (PDO) culture model

In this study, a PDO model was established using tumour tissues from CRC patients at the Department of Gastrointestinal Surgery of the Affiliated Hospital of Xuzhou Medical University. Briefly, freshly removed tumour tissues were cut into 1–3 mm^3^ pieces, washed several times with antibiotic-containing PBS, and digested with 200 U/ml collagenase (Sigma, MO, USA) and 100 U/ml hyaluronidase (Sigma, MO, USA) for 30 min at 37℃. The digested tissues were filtered through a 100 μm cell filter and then centrifuged to discard the supernatant, after which the cells were resuspended in Matrigel. Then, 50 µL of Matrigel resuspension solution was added to each well of a 48-well plate, and 450 µL of CRC organic medium (bioGenous, Suzhou, China) was added to each well after solidification. The medium was changed every 2–3 days, and the organoids were passaged every 10–15 days. Lentiviral transfection was performed 24 h after organoid passaging. Organoid images were obtained with an Olympus FSX100 microscope (Olympus, Tokyo, Japan).

### Animal experiments

Female BALB/c nude mice ranging from 4 to 6 weeks old were obtained from Beijing Vital River Laboratory Animal Technology Co., Ltd. (Beijing, China), and housed under specific pathogen-free conditions. To construct a xenograft tumorigenesis model, transfected HCT116 cells were injected subcutaneously into the axillary region of mice (5 × 10^6^ cells). Tumour growth was measured every 3 days, and the tumour volume (V) was calculated according to the following formula: V= (length × width^2^)/2. When the maximum tumour length diameter reached 15 mm, the mice were sacrificed, and the subcutaneous tumours were removed to calculate the mass and then subjected to HE and IHC staining. The Animal Care and Use Committee of Xuzhou Medical University provided a statement of ethical approval for all animal experiments in this study.

### Statistical analysis

Statistical analyses were performed using SPSS 19.0 software (IBM, Armonk, NY, USA) and GraphPad Prism version 8.0 (La Jolla, CA, USA). All the data in the present study is presented as the means ± standard deviations (SD), and all the tests were two-sided. A *P* value < 0.05 was considered wo indicate statistical significance. Student’s t-test or one-way analysis of variance (ANOVA) was used to evaluate the significance of differences between groups. Correlations were detected by Spearman’s correlation coefficient. The relationship between POU6F2-AS1 expression and the clinicopathological parameters of CRC patients was calculated by the chi-square test or Fisher’s exact test. Overall survival (OS) was assessed by the Kaplan–Meier method and log-rank test. Univariate and multivariate Cox proportional hazard regression models were used to evaluate the effects of POU6F2-AS1 expression or other clinicopathological parameters on survival and the hazard ratio (HR).

### Additional methods

Additional experiments, including cell transfection, RNA extraction, qRT–PCR, immunohistochemistry (IHC), RNA stability assays, fluorescence in situ hybridization (FISH) and immunofluorescence (IF) staining, Western blotting, Cell Counting Kit-8 (CCK–8) assays, 5-ethynyl-2’-deoxyuridine (EdU) assays, colony formation assays, methylated RNA Immunoprecipitation (MeRIP)-qPCR, RNA immunoprecipitation (RIP)-qPCR, RNA sequencing and bioinformatics analysis, application of palmitic acid (PA) and orlistat, and liquid chromatography mass spectrometry (LC-MS) based FA analysis are described in Additional file 2: Supplemental Materials and Methods.

## Results

### Identification of POU6F2-AS1 and analysis of its clinical significance in CRC

**Fig. 1:**
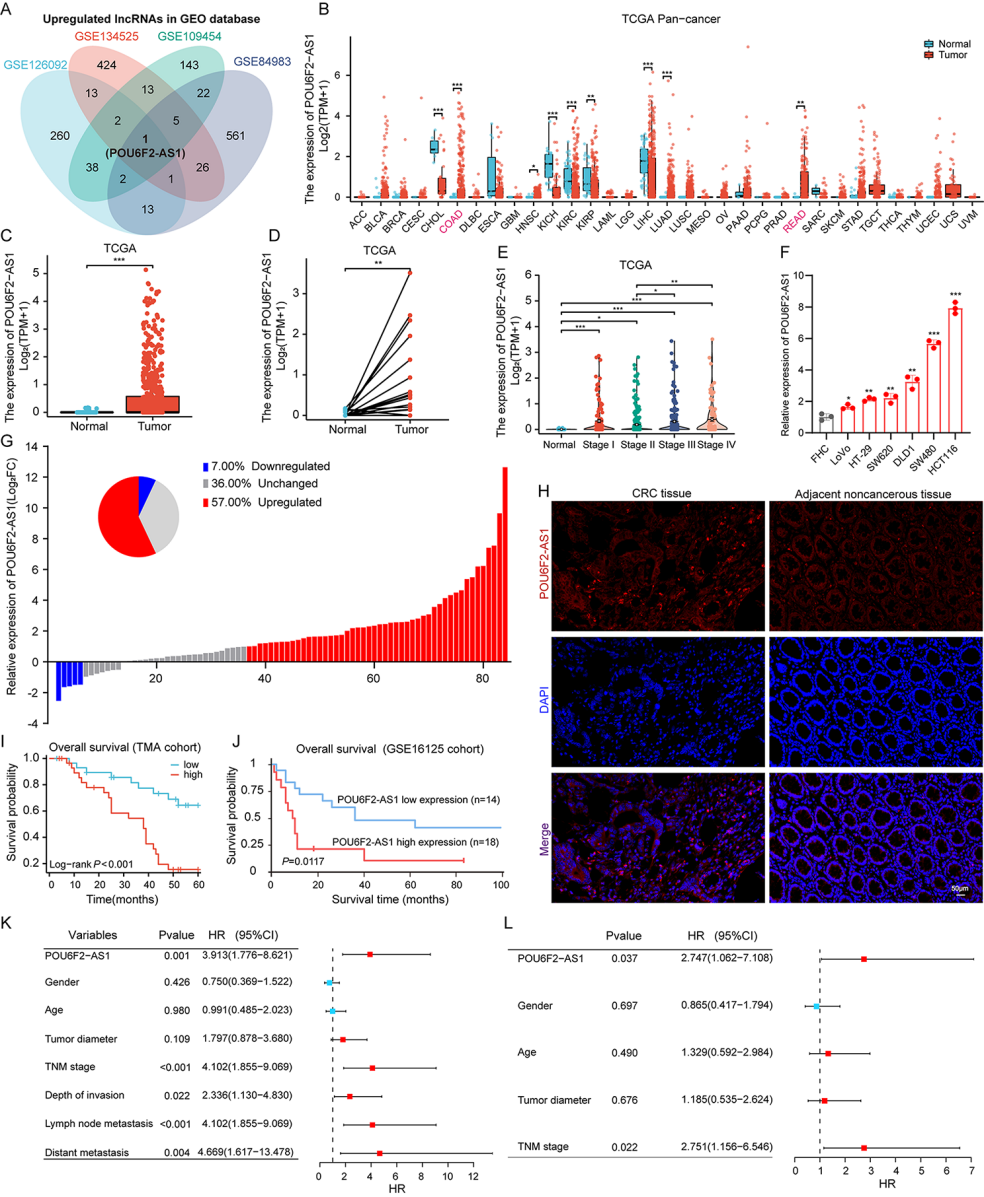
Identification of POU6F2-AS1 and analysis of its clinical significance in CRC. (**A**) Venn diagram for upregulated lncRNA in GEO datasets. (**B**) Comparison of POU6F2-AS1 expression status between different cancers or specific cancer subtypes and normal tissues in TCGA database. (**C**) The POU6F2-AS1 expression between CRC tissues and normal samples in TCGA database. (**D**) The POU6F2-AS1 expression between CRC tissues and paired normal samples in TCGA database. (**E**) The expression of POU6F2-AS1 was evaluated in different groups stratified according to clinical stages. (**F**) Relative expression of POU6F2-AS1 in a human normal colorectal epithelium cell line (FHC) and CRC cell lines (HCT116, SW480, DLD1, SW620, HT29 and LoVo). (**G**) Comparison of the relative POU6F2-AS1 expression between CRC tissues and paired ANTs (case = 84). (**H**) Representative images showing the expression of POU6F2-AS1 in CRC TMAs detected by FISH staining. (**I**) Kaplan-Meier analysis of the OS rate in CRC patients according to POU6F2-AS1 expression based on TMAs cohort (*P* < 0.001). (**J**) The OS survival curves of POU6F2-AS1 in GSE16125 database (*P* = 0.0117). (K, L) Univariate and Multivariate analyses were performed for CRC patients in TMAs. All bars correspond to 95% CIs. HR, hazard ratio CI, confidence interval. CRC, colorectal cancer; AUC, area under the curve. *P < 0.05, **P < 0.01, ***P < 0.001

To identity novel oncogenic lncRNAs that are involved in the tumorigenesis and progression of CRC, four publicly available datasets (GSE126092, GSE134525, GSE109454, and GSE84983) were downloaded from the Gene Expression Omnibus (GEO) database for analysis (Fig. S1A-D). Overall, differential expression analysis of these datasets was performed with thresholds of |log FC| ≥2 and *P* value < 0.05, and a Venn diagram of the differentially expressed genes (DEGs) revealed that only the lncRNA POU6F2-AS1 was significantly upregulated in all four datasets (Fig. 1A). Next, the expression level of POU6F2-AS1 was detected in The Cancer Genome Atlas (TCGA) database. Given that POU6F2-AS1 is a novel lncRNA that has not been well characterized, we first analyzed the expression of POU6F2-AS1 in various types of cancer using the TCGA database. According to the results of the pan-cancer analysis, compared with those in corresponding normal tissues, the expression levels of POU6F2-AS1 were significantly dysregulated in many solid cancers, including colon adenocarcinoma (COAD) and rectum adenocarcinoma (READ) (Fig. 1B). Next, we narrowed our focus and performed on the comprehensive analysis of the expression and clinical significance of POU6F2-AS1 in CRC (COAD and READ in TCGA databases). The POU6F2-AS1 expression level in CRC tissues was significantly greater than that in normal tissues and was positively associated with advanced TNM stage (Fig. 1C-E). Moreover, analysis of data from the Cancer Cell Line Encyclopedia (CCLE) database revealed that the POU6F2-AS1 expression level in bowel cells was greater than that in most of the other cell lines from different tissues (Fig. S1E). Similar results were observed in CRC cell lines, POU6F2-AS1 was significantly upregulated in HCT116, SW480, DLD1, SW620, HT29 and LoVo CRC cells compared with FHC cells (human normal colorectal mucosal cells) (Fig. 1F).

To verify the POU6F2-AS1 expression pattern in CRC tissues, we first evaluated its expression in 84 paired fresh frozen tissue specimens using quantitative real–time polymerase chain reaction (qRT–PCR). qRT–PCR analysis revealed that the POU6F2-AS1 expression level was significantly greater in CRC tissues than in adjacent normal tissues (ANTs) (Fig. 1G). Moreover, fluorescence in situ hybridization (FISH) assays of a tissue microarray (TMA) of 60 pairs of CRC tissues and ANTs stained with the POU6F2-AS1 probe also showed that the expression of POU6F2-AS1 was significantly upregulated in CRC tissues (Fig. 1H, Fig. S1F, G). The results of qRT–PCR and FISH analyses were consistent with those of the bioinformatic analysis of the GEO and TCGA databases.

To further characterize the clinical significance of POU6F2-AS1 in the TMA cohort, a chi-square test was performed to analyse the relationship between POU6F2-AS1 expression and clinicopathological features. The results showed that POU6F2-AS1 expression was positively associated with tumour diameter (*P* = 0.038), depth of invasion (*P* < 0.001), lymph node metastasis (*P* = 0.010), and TNM stage (*P* = 0.010) (Additional file 1: Table S1). In addition, Kaplan–Meier (K–M) survival curve analysis and Cox regression analysis were performed to analyse the prognostic value of POU6F2-AS1 in CRC. K–M survival curves revealed that a higher level of POU6F2-AS1 expression was associated with poor overall survival (OS) in CRC patients in the TMA cohort (Fig. 1I). A similar result was verified in another cohort via analysis of the corresponding the GSE16125 dataset (Fig. 1J). Univariate Cox regression analysis suggested that POU6F2-AS1 expression, TNM stage, depth of invasion, lymph node metastasis, and distant metastasis were significant risk factors in terms of OS (Fig. 1K), while multivariate Cox analysis showed that POU6F2-AS1 expression and TNM stage could serve as independent prognostic factors in terms of OS in CRC patients (Fig. 1L).

### POU6F2-AS1 promotes the growth of CRC cells and PDO in vitro

**Fig. 2:**
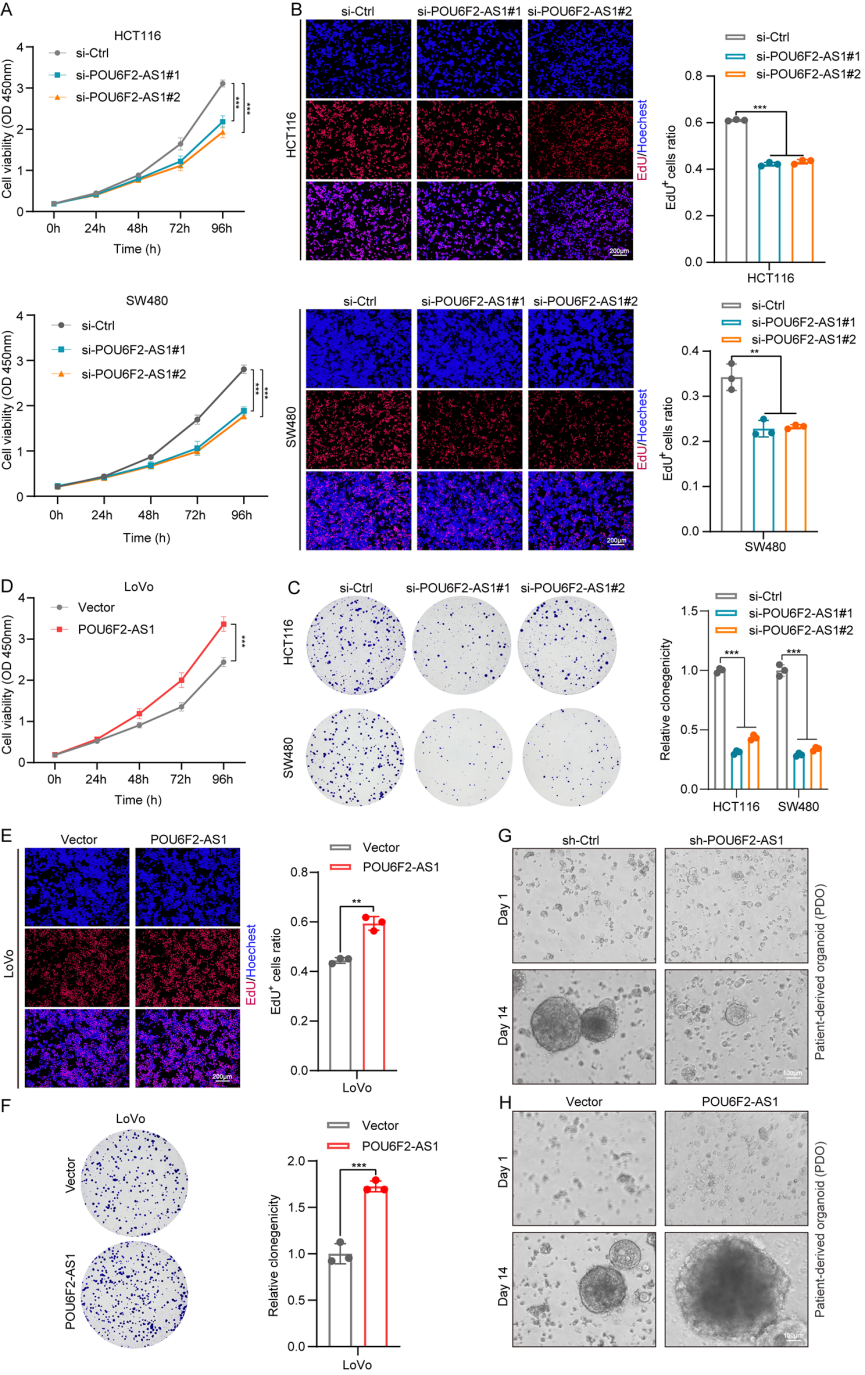
POU6F2-AS1 promotes the growth of CRC cells and PDO in vitro. (**A**, **D**) The viability curves of CRC cells with knockdown or overexpression of POU6F2-AS1 were detected by CCK–8 assays at the indicated time points. (**B**, **E**) EdU assays were performed to assess the proliferation ability of CRC cells with POU6F2-AS1 knockdown or overexpression. (**C**, **F**) Colony formation assays were conducted to determine the proliferation of CRC cells transfected with POU6F2-AS1 knockdown or overexpression. (**G**, **H**) CRC patient-derived organoid was infected with POU6F2-AS1 overexpression or knockdown or a control lentivirus, and representative bright-field images are exhibited. The data are presented as the mean ± SD. *P < 0.05, **P < 0.01, ***P < 0.001

The previous analysis of endogenous POU6F2-AS1 expression in CRC cell lines revealed significant upregulation in HCT116 and SW480 cells and relatively low expression in LoVo cells (Fig. 1F). To determine the biological function of POU6F2-AS1 in CRC cells, we constructed two siRNAs (si-POU6F2-AS1#1 and si-POU6F2-AS1#2) and one shRNA (sh-POU6F2-AS1) targeting POU6F2-AS1 and one overexpression plasmid (POU6F2-AS1). Subsequently, we knocked down POU6F2-AS1 in HCT116 and SW480 cells and overexpressed it in LoVo cells (Fig. S1H–J). CCK–8, EdU and colony formation assays revealed that POU6F2-AS1 knockdown significantly suppressed the growth of CRC cells, whereas POU6F2-AS1 overexpression had the opposite effect (Fig. 2A–F). Furthermore, we generated patient-derived organoid (PDO) from CRC patient tissues and found that POU6F2-AS1 knockdown suppressed PDO formation, while POU6F2-AS1 overexpression facilitated PDO formation (Fig. 2G, H). These results clearly demonstrated that POU6F2-AS1 promotes the proliferation of CRC cells and PDO.

### POU6F2-AS1-mediated de novo lipogenesis is essential for CRC cell and PDO growth in vitro

**Fig. 3:**
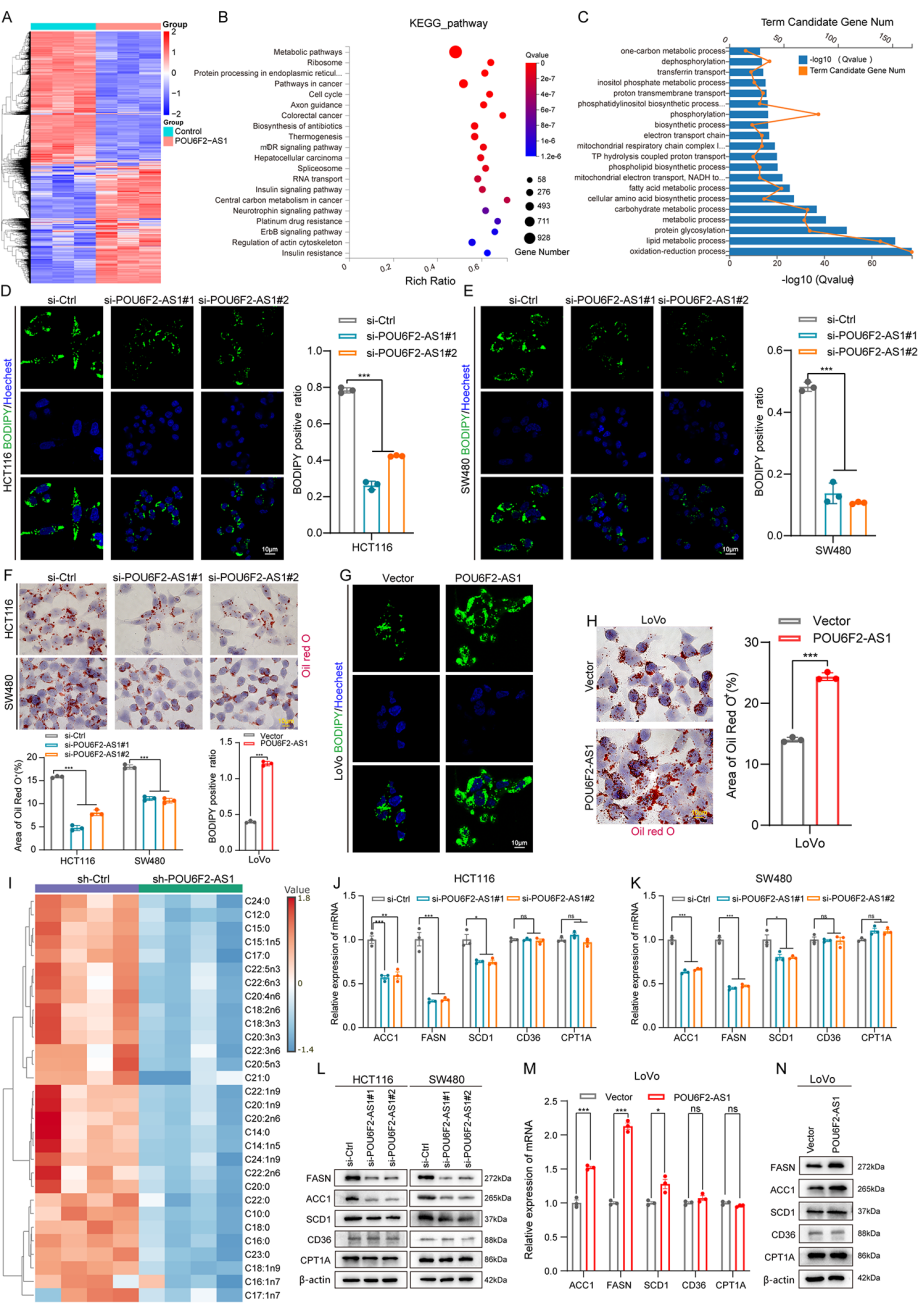
POU6F2-AS1 facilitates de novo lipogenesis of CRC cell and PDO growth in vitro. (**A**) Heatmap of the differentially expressed genes after POU6F2-AS1 overexpression in CRC cells. (**B**) KEGG enrichment analysis showed the significantly enriched pathways after POU6F2-AS1 overexpression. (**C**) Gene Ontology (GO) analysis of these differentially expressed genes (DEGs) enriched in metabolic process showed the top 20 significantly enriched terms of biological process. (**D, E, G**) Representative confocal microscopy images of intracellular lipid droplets in CRC cells with POU6F2-AS1 knockdown or overexpression stained with BODIPY 493/503 dye (green). Nuclei were stained with DAPI (blue). (**F, H**) Representative images of intracellular lipid droplets in CRC cells with POU6F2-AS1 knockdown or overexpression stained with Oil Red O. (**I**) Heatmap showing liquid chromatography mass spectrometry (LC-MS) based FA analysis of HCT116 cells bearing control or POU6F2-AS1 knockdown. (**J-N**) qRT–PCR and western blotting analysis for mRNA and protein expression levels of key enzymes involved in FA synthesis (FASN, ACC1, and SCD1), FA β-oxidation (CPT1A) and FA uptake catabolism (CD36) in CRC cells with POU6F2-AS1 knockdown or overexpression. The data are presented as the mean ± SD. *P < 0.05, **P < 0.01, ***P < 0.001

To understand the underlying biological pathways by which POU6F2-AS1 facilitates CRC tumorigenesis and growth, we performed RNA-seq on CRC cells transfected with the POU6F2-AS1 overexpression plasmid or empty vector and found a total of 2030 differentially expressed genes (DEGs) (*P* < 0.05, |log FC| ≥ 1) were detected (Fig. 3A). KEGG enrichment analysis revealed that the significantly enriched pathways included metabolic pathways, pathways in cancer, cell cycle, colorectal cancer, central carbon metabolism in cancer (Fig. 3B). Gene ontology biological process (GO-BP) enrichment analysis also revealed that the DEGs between POU6F2-AS1 high and low groups were associated metabolic processes, biological regulation, cell proliferation, growth, etc. (Fig. S1K). To gain further insight into the molecular mechanism of POU6F2-AS1 involved in the regulation of metabolic pathways, Gene Ontology (GO) analysis of the DEGs enriched in metabolic processes revealed that the top 20 significantly enriched terms associated with biological processes included lipid metabolic process and FA metabolic process (Fig. 3C).

Abnormal lipid metabolism reprogramming can provide abundant nutrients for tumorigenesis and cancer progression [19]. Therefore, we sought to explore the role of POU6F2-AS1 in the aberrant lipid metabolism of CRC cells. Intracellular staining with the lipophilic fluorescent dye BODIPY 493/503 and Oil Red O and an intracellular triglyceride (TAG) assay indicated that POU6F2-AS1 knockdown decreased lipid stores and TAG content, while POU6F2-AS1 overexpression increased lipid stores and TAG contents in CRC cells (Fig. 3D–H; Fig. S2A–C). More importantly, liquid chromatography–mass spectrometry (LC-MS) revealed that knockdown of POU6F2-AS1 significantly decreased FAs levels in CRC cells, such as palmitic acid (C16:0), stearic acid (C18:0), palmitoleic acid (C16:1), and oleic acid (C18:1) (Fig. 3I). These data demonstrate that that POU6F2-AS1 may facilitate the accumulation of lipids by positively regulating FA metabolism.

The increased FA metabolism of cancer cells could be achieved through de novo FA synthesis, FA uptake and decreased FA catabolism [20, 21]. To clarify how POU6F2-AS1 regulate FA metabolism, we detected the effect of POU6F2-AS1 on the expression levels of key enzymes involved in FA synthesis (FASN, ACC1, and SCD1), FA β-oxidation (CPT1A) and FA uptake catabolism (CD36). The results showed that the knockdown of POU6F2-AS1 significantly reduced the relative expression of FASN, ACC1, and SCD1 at both the mRNA and protein levels, while had no effect on the expression of CPT1A and CD36 (Fig. 3J-L). Consistently, overexpression of POU6F2-AS1 remarkably increased the mRNA and protein levels of the FA synthetic enzymes in CRC cells (Fig. 3M, N), demonstrating that POU6F2-AS1 promoted de novo FA synthesis of CRC cells.

We further evaluated whether the POU6F2-AS1-mediated modulation of FA metabolism is essential for CRC cell growth. We used orlistat, a FA synthase inhibitor, to decrease the lipid content in CRC cells overexpressing POU6F2-AS1; on the other hand, we used palmitic acid (PA) to increase the lipid content in CRC cells with POU6F2-AS1 knockdown. The results showed that orlistat treatment attenuated the effects of POU6F2-AS1 overexpression on cell growth, whereas PA treatment rescued the effects exerted by POU6F2-AS1 knockdown on cell growth (Fig. S2D-K). These findings confirmed the fundamental roles of POU6F2-AS1-mediated FA accumulation in driving CRC cell growth.

### POU6F2-AS1 interacts with YBX1 to promote CRC cell growth and lipogenesis

**Fig. 4:**
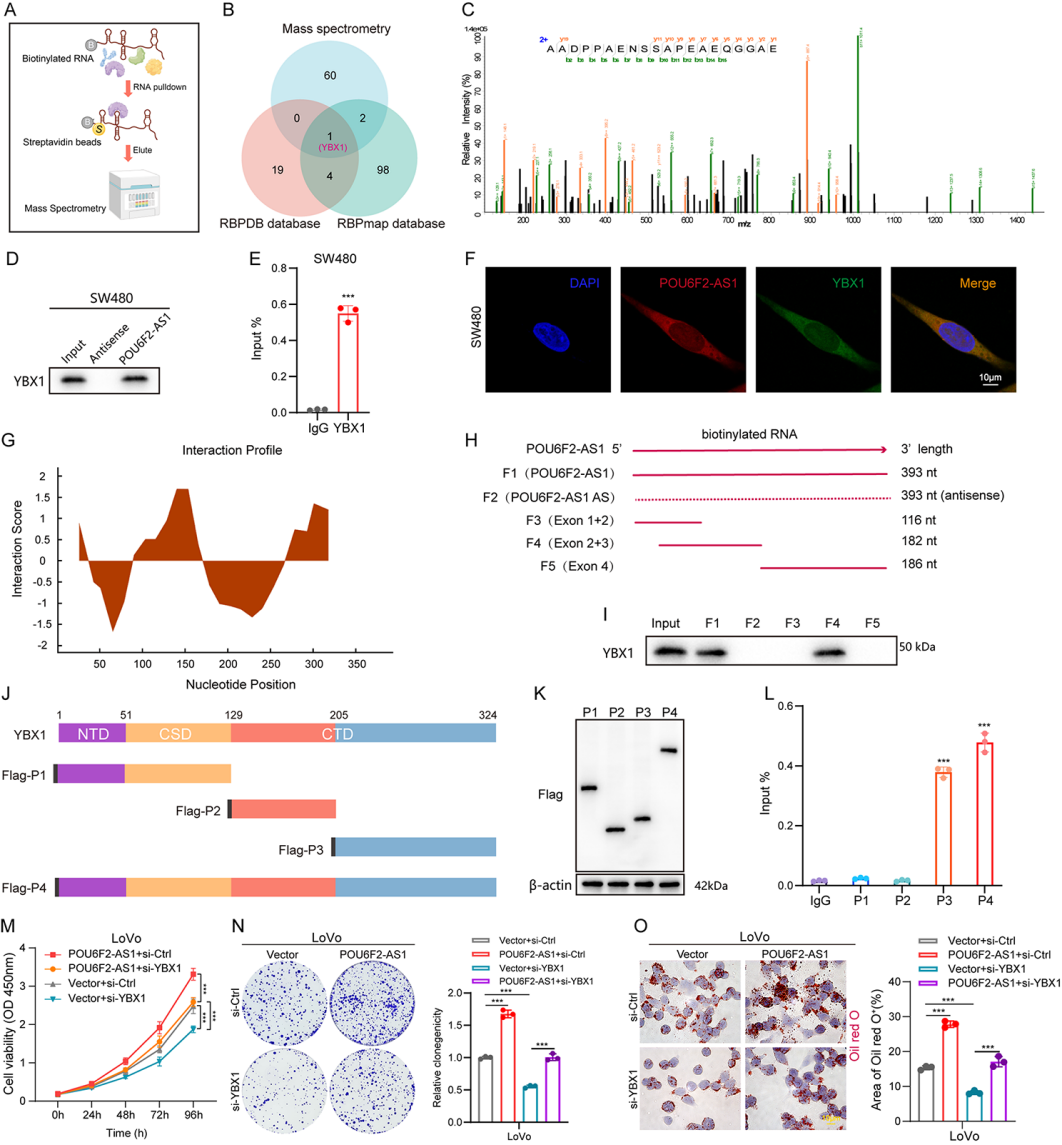
POU6F2-AS1 interacts with YBX1 to promote CRC cell growth and lipogenesis. (**A**) Schematic workflow of the biotinylated RNA pull-down assay and Mass spectrometry used for identification of POU6F2-AS1 binding proteins. (**B**) Venn diagram exhibiting the proteins pulled down by biotin-labeled POU6F2-AS1, and overlapping analysis with RBPDB and RBPmap database. (**C**) Representative YBX1 peptides binding with POU6F2-AS1 were detected by MS. (**D**) Western blotting of YBX1 pull downed by biotin-labeled POU6F2-AS1 was shown. (**E**) qRT–PCR analysis of POU6F2-AS1 enriched by YBX1 protein in SW480 cells was shown. (**F**) Colocalization analysis was assessed with the specific probes against POU6F2-AS1 (red) and the specific antibody against YBX1 (green). Nuclei are stained with DAPI (blue). (**G**) The potential interaction regions of POU6F2-AS1 with YBX1 were predicted by catRAPID website. (**H**) Schematic view of truncated fragments of POU6F2-AS1. (**I**) Western blotting analysis of YBX1 in samples pulled down by biotin-labeled full-length (F1, 2) or truncated POU6F2-AS1 (F3–F5). (**J**) Domain mapping of Flag-labeled full-length YBX1 (P4) or truncated YBX1 (P1–P3). (**K**) Western Blot analysis of Flag-tagged YBX1 constructs in SW480 cells transfected with each construct (P1: 1–129 aa, P2: 130–205 aa, P3: 206–324 aa). (**L**) RIP assays were performed by the antibodies against Flag, followed by POU6F2-AS1 qRT–PCR in SW480 cells. (**M**) CCK–8 assay in POU6F2-AS1 overexpression LoVo cells with YBX1 knockdown. (**N**) Colony formation assay in POU6F2-AS1 overexpression LoVo cells with YBX1 knockdown. (**O**) Oil Red O staining assay in POU6F2-AS1 overexpression LoVo cells with YBX1 knockdown. The data are presented as the mean ± SD. *P < 0.05, **P < 0.01, ***P < 0.001

The functions of lncRNAs are tightly related to their subcellular location. To further clarify the molecular mechanism of POU6F2-AS1 in CRC, FISH was performed, and the results showed that POU6F2-AS1 was located in both the nucleus and cytoplasm (Fig. S3A). Accumulating evidence has demonstrated that interactions of lncRNAs with proteins play vital roles in cancer progression [22–24]. Intriguingly, our GO molecular function (GO-MF) analysis revealed that the top enriched term was binding (Fig. S1K), which indicated that POU6F2-AS1 may exert its effects by binding to specific protein(s). We subsequently screened potential POU6F2-AS1-binding protein(s) via biotin-labelled RNA pull-down and subsequent mass spectrometry (MS) analyses, and approximately 64 potential interacting proteins were pulled down with POU6F2-AS1 in HCT116 cells (Fig. 4A and Additional file 1: Table S2). Overlapping analysis of RNA-binding proteins (RBPs) predicted by the online RBPDB database and RBPmap database revealed that only YBX1 was the potential partner of POU6F2-AS1 (Fig. 4B, C).

YBX1 is a DNA/RNA binding protein that dysregulates a wide range of genes involved in cancer apoptosis, proliferation, differentiation, and drug resistance [25]. Recent studies have indicated that YBX1 is upregulated in CRC and drives CRC progression [26–28]. Correlation analysis according to data from the TCGA database indicated that the expression of POU6F2-A1 was positively associated with that of YBX1 in CRC (Fig. S3B). Next, biotin-labelled RNA pull-down and RNA immunoprecipitation (RIP) and assays were used to validate the physical interaction between POU6F2-AS1 and YBX1 (Fig. 4D, E). FISH and immunofluorescence (IF) colocalization assays showed that POU6F2-AS1 and YBX1 colocalized in the nucleus and cytoplasm of CRC cells (Fig. 4F). Furthermore, the catRAPID analysis and representative YBX1 peptide detected by MS revealed a potential interaction region between POU6F2-AS1 and YBX1 (Fig. 4G, C). Moreover, the secondary structure of POU6F2-AS1 was predicted via RNAfold software (Fig. S3C). To further elucidate the binding regions between POU6F2-AS1 and YBX1, we constructed one biotinylated full-length POU6F2-AS1 construct and four biotinylated fragments of POU6F2-AS1 (F1: full-length sense of POU6F2-AS1; F2: full-length antisense of POU6F2-AS1; F3: Exon1 + 2, 1–116 nt; F4: Exon2 + 3, 14–195 nt; F5: Exon4, 196–381 nt) for RNA pull-down experiments with CRC cell lysates (Fig. 4H). Deletion mapping analysis revealed that the 117–195 nt region of POU6F2-AS1 was indispensable for its interaction with YBX1 (Fig. 4I). As previously reported, YBX1 consists of three domains, the N-terminal domain (NTD), the cold shock domain (CSD), and the large C-terminal domain (CTD) (Fig. 4J) [29]. To identify the domain involved in the binding of YBX1 to POU6F2-AS1, we cloned a series of Flag-tagged truncated YBX1 proteins (P1: 1–129 aa; P2: 130–205 aa; P3: 206–324 aa; and P4: 1–324 aa) (Fig. 4K). RIP assays were conducted with an anti-Flag antibody, and the results revealed that both the P3 domain and the P4 domain specifically interact with POU6F2-AS1 (Fig. 4L), suggesting that amino acids 206–324 in the CTD domain of YBX1 are required for the interaction with POU6F2-AS1.

Next, we characterized the role of POU6F2-AS1/YBX1 regulatory axis in CRC. First, we confirmed that YBX1 expression was markedly upregulated in CRC samples based on analysis of the TCGA database (Fig. S3D, E). Afterwards, knockdown of YBX1 by specific siRNA was validated by qRT–PCR and Western blotting (Fig. S3F, G). We knockdown YBX1 in LoVo cells with stable overexpression of POU6F2-AS1 and observed that POU6F2-AS1 overexpression promoted CRC cell growth and lipogenesis; these effects were attenuated by YBX1 knockdown (Fig. 4M-O). Consistently, the decreases of size of PDO and suppression of CRC cell growth and lipogenesis caused by POU6F2-AS1 knockdown were restored by YBX1 overexpression (Fig. S3H-J). Overall, these findings suggested that YBX1 plays a carcinogenic role in CRC cells and that POU6F2-AS1 may drive the growth and lipogenesis of CRC cells by directly binding to YBX1.

### POU6F2-AS1 tethers YBX1 to the FASN promoter to transcriptionally activate FASN

**Fig. 5:**
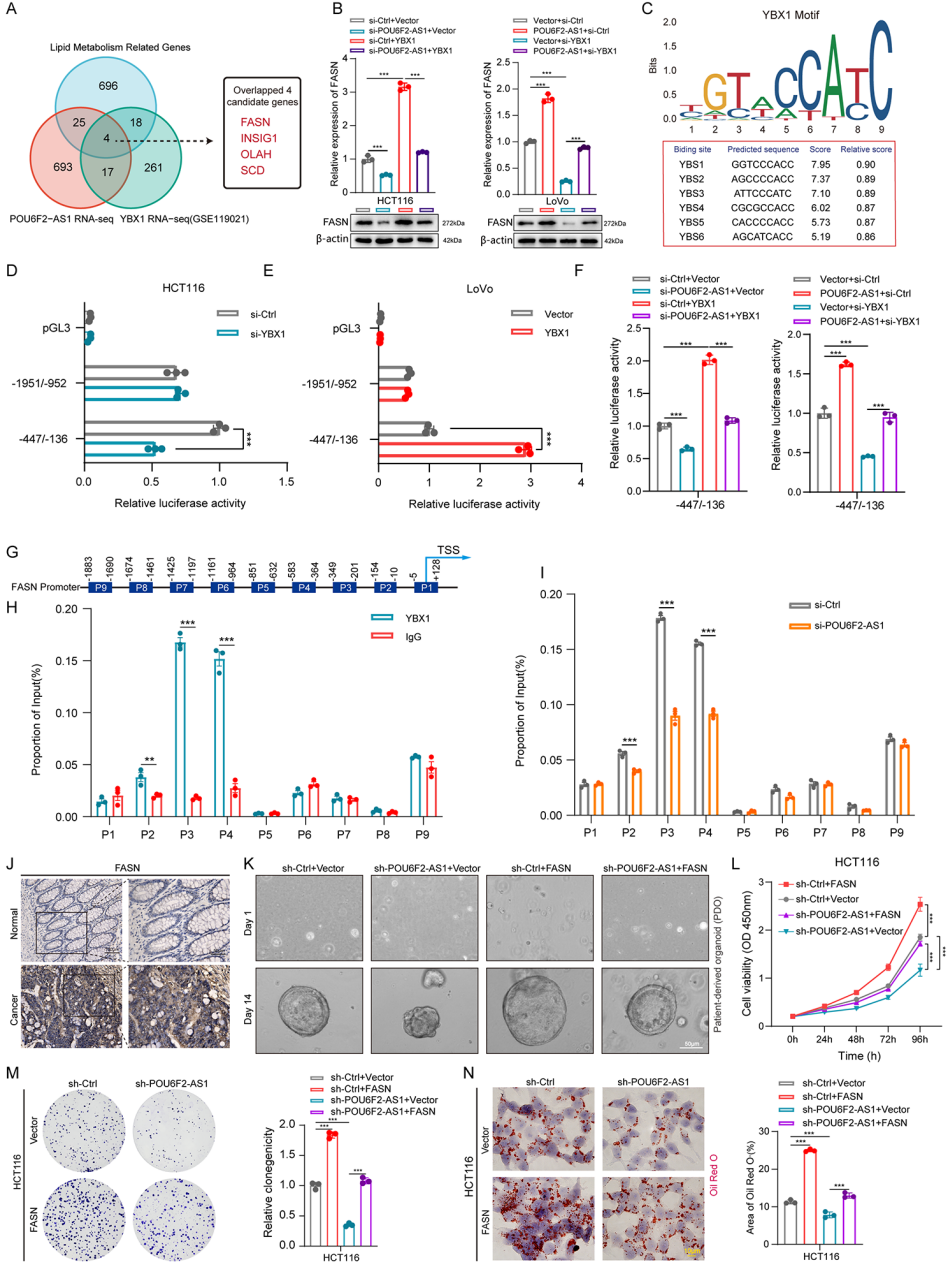
POU6F2-AS1 tethers YBX1 to the FASN promoter to transcriptionally activate FASN and promotes CRC cell progression. (**A**) Venn diagram exhibiting the downstream genes coregulated by POU6F2-AS1 and YBX1, and overlapping analysis with lipid metabolism-related genes (LMGs). (**B**) qRT–PCR and Western blotting showed that the reduction in FASN in HCT116 cells with transfected with POU6F2-AS1 siRNAs was reversed by YBX1 overexpression, whereas the increase in FASN in LoVo cells transfected with POU6F2-AS1 overexpression plasmid was reversed by YBX1 knockdown. (**C**) The YBX1-binding motif and sites enriched in the FASN promoter was predicted by JASPAR website. (**D**, **E**) Luciferase reporter assays showed that the luciferase activity driven by the − 447/-136 fragment of the FASN promoter region was significantly reduced in HCT116 cells with YBX1 knockdown but enhanced in LoVo cells with YBX1 overexpression. (**F**) Luciferase reporter assays for − 447/-136 fragment of the FASN promoter activity in SW480 cells with POU6F2-AS1 knockdown with or without overexpressing YBX1 (left), and in LoVo cells with POU6F2-AS1 overexpression with or without knocking down YBX1 (right). (**G**) The schematic diagram of truncated FASN promoter. (**H**) ChIP-qPCR analysis was performed to determine the binding affinity of YBX1 to nine FASN promoter regions in SW480 cells, showing that YBX1 bound to P2-4 regions in the FASN promoter. ChIP-qPCR with IgG was used as the negative control. (**I**) ChIP-qPCR analysis for the enrichment of YBX1 at the FASN promoter regions in SW480 cells with PO6F2-AS1 knockdown. (**J**) Representative images showing the expression of FASN in CRC tissues and paired ANTs detected by IHC. (**K**) CRC patient-derived organoid infected with POU6F2-AS1 knockdown or FASN overexpression lentivirus. (**L**) CCK–8 assay in POU6F2-AS1 knockdown HCT116 cells with FASN overexpression. (**M**) Colony formation assay in POU6F2-AS1 knockdown HCT116 cells with FASN overexpression. (**N**) Oil Red O staining assay in POU6F2-AS1 knockdown HCT116 cells with FASN overexpression. The data are presented as the mean ± SD. *P < 0.05, **P < 0.01, ***P < 0.001

To further elucidate the mechanism by which the POU6F2-AS1/YBX1 axis drives oncogenesis and lipogenesis in CRC and to identify the downstream target of the axis, we assessed the lipid metabolism-related genes (LMGs) coregulated by POU6F2-AS1 and YBX1. A Venn diagram revealed that four genes (FASN, INSIG1, OLAH, and SCD) were potential targets of the POU6F2-AS1/YBX1 axis (Fig. 5A). FASN is a key enzyme in lipid metabolism, especially in the de novo synthesis of FAs. Upregulated FASN expression has been reported to be associated with cell proliferation in multiple cancer types, including CRC [10, 30, 31]. In addition, Wang et al. reported that YBX1 depletion repressed the expression of FASN in glioblastoma cells, but the involved regulatory mechanism is still unknown [32]. Hence, we focused on the role of FASN in POU6F2-AS1/YBX1 axis-dependent CRC progression.

Correlation analysis according to the TCGA database demonstrated that FASN expression was positively associated with POU6F2-AS1 and YBX1 expression in CRC (Fig. S4A, B). Next, we determined whether POU6F2-AS1 could regulate the expression of FASN by binding to YBX1. Western blotting and qRT–PCR assays revealed that the decrease in FASN levels caused by POU6F2-AS1 knockdown could be reversed by YBX1 overexpression in HCT116 cells, while the increase in FASN levels induced by POU6F2-AS1 overexpression was attenuated by YBX1 knockdown in LoVo cells (Fig. 5B).

Next, we further investigated the regulatory mechanism by which the POU6F2-AS1/YBX1 axis enhances the FASN expression. GO analysis revealed that the most significantly enriched molecular function included transcription regulator activity (Fig. S1K). YBX1 is a well-established transcription factor that has been reported to induce the transcription of oncogenic genes, such as FOXA1 [33], FZD7 [34], and HOXC8 [35] in cancers. Intriguingly, previous studies have reported that transcriptional regulation is one of the most important mechanisms of FASN overexpression [36]. We used the JASPAR website to predict the underlying binding site (s) for YBX1 in the FASN promoter region and found 6 high-confidence response elements located within the FASN promoter region, these were named YBS1-6 (Fig. 5C). These data indicated that POU6F2-AS1 might exert its effects by recruiting YBX1 to the FASN promoter to induce transcriptional activation.

To evaluate whether YBX1 transcriptionally activates FASN, two different fragments of the FASN promoter were cloned and inserted into luciferase reporter vectors. The results of luciferase reporter assays showed that the luciferase activity of the reporter containing the − 447/-136 fragment upstream of the FASN transcription start site (TSS) was significantly decreased in HCT116 and SW480 cells with YBX1 knockdown but increased in cells with YBX1 overexpression (Fig. 5D, E; Fig. S4C). Moreover, neither knockdown nor overexpression of YBX1 altered the luciferase activity of the reported containing the − 1951/-952 fragment (Fig. 5D, E; Fig. S4C). These results confirmed that the YBX1 binding site (s) were located within the − 447/-136 region of the FASN promoter. To further determine whether POU6F2-AS1 activates FASN by recruiting YBX1 to its promoter, we detected the transcriptional activity of the FASN promoter after altering the expression of POU6F2-AS1. Luciferase assays showed that overexpressing YBX1 in POU6F2-AS1 knockdown cells markedly restored FASN promoter activation. On the other hand, knockdown of YBX1 in POU6F2-AS1 overexpressing cells significantly attenuated FASN promoter activation (Fig. 5F). To further clarify which specific site (s) YBX1 binds within the FASN promoter, 9 pairs of primers (denoted P1–9) were designed for different regions of the FASN promoter (Fig. 5G). Chromatin immunoprecipitation (ChIP) assays revealed that YBX1 binds to P2–4 regions of the FASN promoter (Fig. 5H). More importantly, knockdown of POU6F2-AS1 decreased YBX1 enrichment in the P2–4 regions of the FASN promoter, indicating that deletion of POU6F2-AS1 significantly decreased the binding of YBX1 to the FASN promoter (Fig. 5I). Taken together, these results show that POU6F2-AS1 tethers YBX1 to the FASN promoter to induce transcriptional activation of FASN.

### FASN is involved in POU6F2-AS1-induced growth and lipogenesis in CRC

To further validate the role of FASN in POU6F2-AS1-mediated CRC progression, bioinformatics analysis was performed. FASN expression was markedly upregulated in CRC tissues vs. normal tissues in the TCGA databases, and these results were confirmed by IHC staining of CRC tissue and ANTs (Fig. S4D, E; Fig. 5J). Next, we constructed siRNA (si-FASN) targeting FASN and one overexpression plasmid (FASN) (Fig. S4F-H). Moreover, rescue experiments showed that the inhibitory effects of POU6F2-AS1 knockdown on the proliferation and lipogenesis of CRC cells and the growth of PDO (indicated by size) were significantly restored by ectopic overexpression of FASN (Fig. 5K-N. Fig. S4I-K). On the other hand, FASN knockdown efficiently attenuated promotion of proliferation and lipogenesis of CRC cells induced by POU6F2-AS1 overexpression (Fig. S4L-N). These results suggested that the promotion of CRC cell growth and lipogenesis by POU6F2-AS1 is partially dependent on FASN.

### POU6F2-AS1 drives the growth and lipogenesis of CRC cells in vivo

**Fig. 6:**
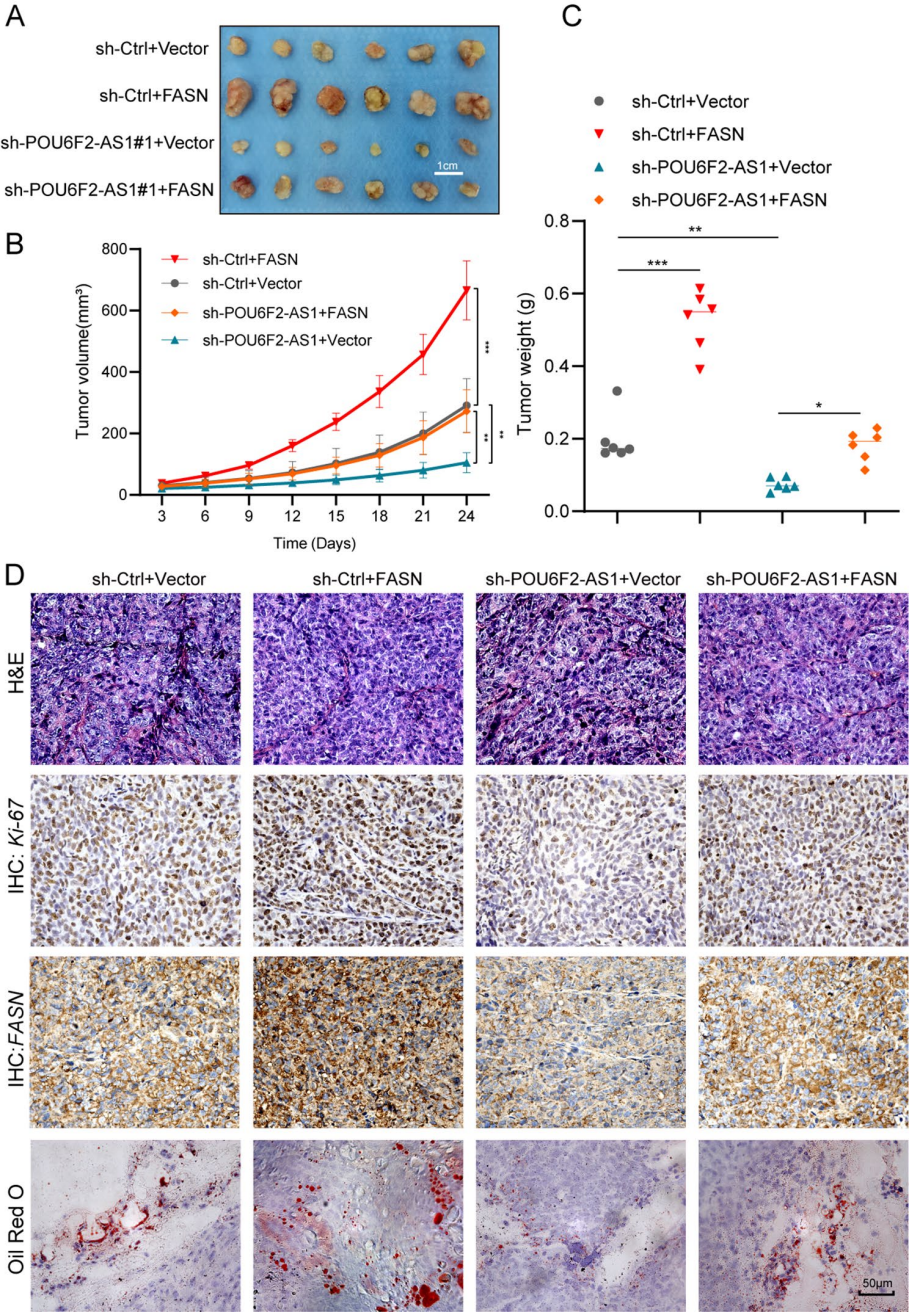
POU6F2-AS1 drives growth and lipogenesis of CRC cells in vivo. (**A**) Image of dissected subcutaneous xenograft tumours from different groups. (**B**, **C**) Growth curve and weight analysis of xenograft tumours in nude mice. (**D**) Representative images of H&E staining, FASN and Ki-67 IHC staining, and Oil Red O staining in different groups of xenograft tumour tissues. *P < 0.05, **P < 0.01, ***P < 0.001

To further verify the in vivo contribution of the POU6F2-AS1/FASN axis to promoting CRC cell growth and lipogenesis, we performed HCT116 cell growth assays and mouse tumour xenograft model experiments. Knockdown of POU6F2-AS1 markedly inhibited cell growth and decreased tumour volume and weight, but FASN overexpression reversed these effects induced by POU6F2-AS1 knockdown, as demonstrated by the increases in cell growth and tumour volume and weight (Fig. 6A-C). Furthermore, IHC and Oil Red O staining revealed that POU6F2-AS1 knockdown decreased FASN protein levels, suppressed tumour cell growth (as measured by Ki-67 staining) and decreased lipid content, while FASN overexpression in these cells restored FASN expression and increased lipogenesis, and elevated Ki-67 staining (Fig. 6D).

### m^6^A modification is involved in the upregulation of POU6F2-AS1 in CRC

**Fig. 7:**
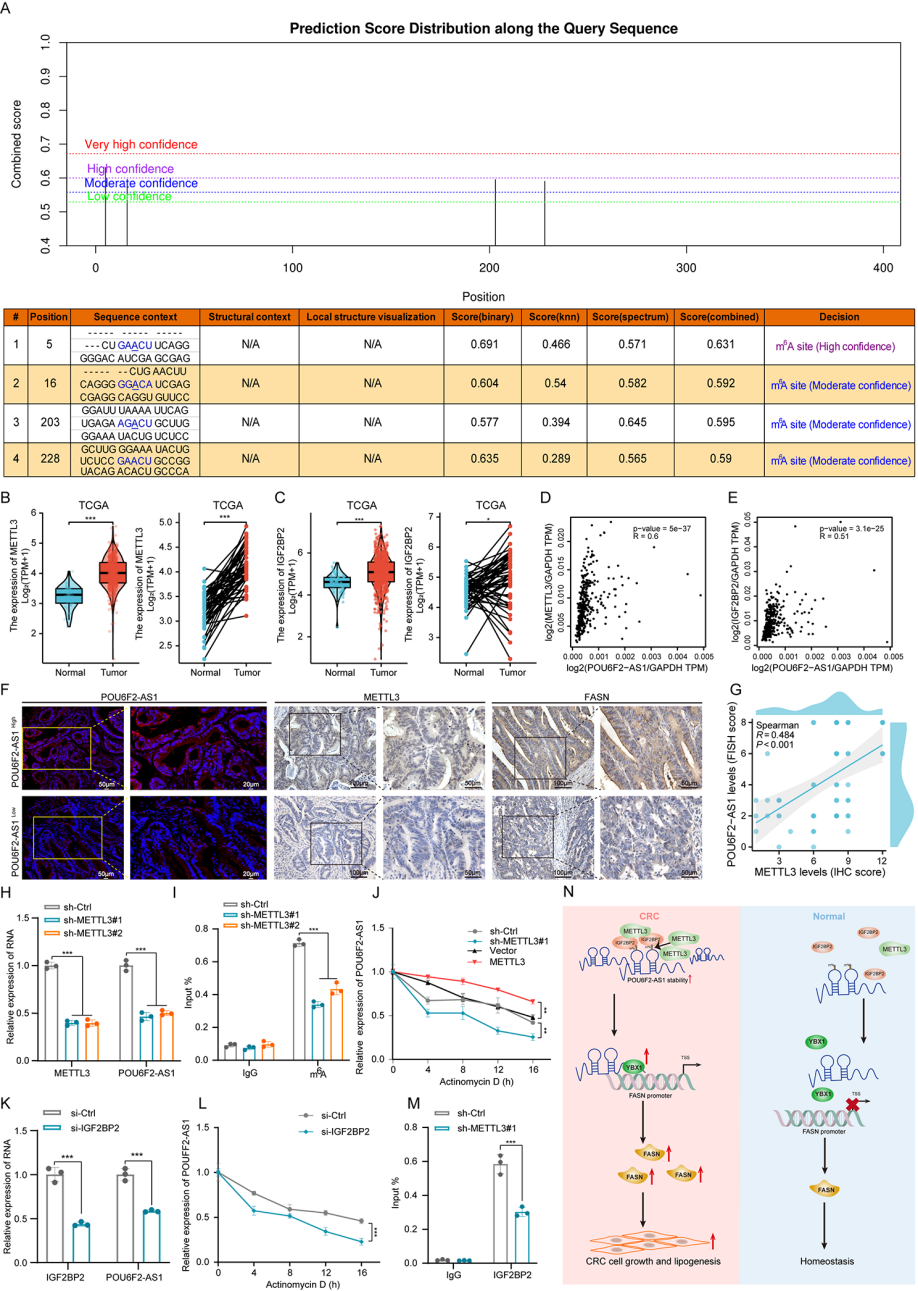
m^6^ A modification is involved in the upregulation of POU6F2-AS1 in CRC. (**A**) The m^6^A modification sites in POU6F2-AS1 were predicted by the SRAMP website. (**B**, **C**) The expression of METTL3 and IGF2BP2 between CRC tissues and normal samples in TCGA database. (**D**, **E**) Correlation of POU6F2-AS1 and METTL3 or IGF2BP2 expression in CRC was analyzed by GEPIA 2 website. (**F**) Representative images of POU6F2-AS1, METTL3 and FASN analyzed by FISH or IHC in POU6F2-AS1 high expression and POU6F2-AS1 low-expression tumours. (**G**) The correlation between POU6F2-AS1 and METTL3 was analyzed in CRC tissues using the FISH score and IHC score. (**H**) After transfecting shRNAs of METTL3 into SW480 cells, the mRNA expression of METTL3 and POU6F2-AS1 was verified by qRT–PCR. (**I**) MeRIP-qPCR analysis of the m^6^A levels in POU6F2-AS1 in SW480 cells transfected with the METTL3 shRNAs. (**J**) The stability of POU6F2-AS1 treatment by actinomycin D at the indicated times following METTL3 knockdown or overexpression in CRC cells was examined by qRT–PCR. (**K**) After transfecting siRNAs of IGF2BP2 into SW480 cells, the mRNA expression of IGF2BP2 and POU6F2-AS1 was verified by qRT–PCR. (**L**) The stability of POU6F2-AS1 treatment by actinomycin D at the indicated times following IGF2BP2 knockdown in SW480 cells was examined by qRT–PCR. (**M**) RIP-qPCR showing the enrichment of IGF2BP2 on POU6F2-AS1 in SW480 cells with METTL3 knockdown. (**N**) Schematic diagram of the mechanism by which the m^6^A modification-mediated POU6F2-AS1 lncRNA facilitates lipogenesis and growth in CRC

Nevertheless, the underlying mechanism leading to the increased expression of POU6F2-AS1 is still unclear. Recent studies have shown that the N6-methyladenosine (m^6^A) modification is involved in the stabilization and expression of lncRNAs in cancer [37, 38]. SRAMP website predictions indicated that several m^6^A sites are dispersed in the POU6F2-AS1 sequence (Fig. 7A). Therefore, we wondered whether the upregulation of POU6F2-AS1 in CRC is dependent on m^6^A modification. Given that methyltransferase-like 3 (METTL3) and insulin like growth factor 2 mRNA binding protein 2 (IGF2BP2) are the key m^6^A methyltransferase “writer” and “reader”, respectively, in CRC cells, we evaluated METTL3 and IGF2BP2 expression and their correlation with POU6F2-AS1 in the TCGA database. Notably, the expression of METTL3 and IGF2BP2 was significantly upregulated in CRC tissues (Fig. 7B, C) and was positively correlated with POU6F2-AS1 expression (*R* = 0.6 and 0.51; Fig. 7D, E). IHC staining of TMAs further confirmed that POU6F2-AS1 expression was positively correlated with METTL3 and FASN expression (Fig. 7F, G). In addition, METTL3 shRNAs and overexpression plasmids were constructed and transfected into cells (Fig. S4O-Q). POU6F2-AS1 expression was apparently reduced with METTL3 knockdown, but increased with METTL3 overexpression (Fig. 7H; Fig. S4Q). MeRIP assays also revealed that METTL3 knockdown reduced the m^6^A modification level of POU6F2-AS1 in SW480 cells (Fig. 7I). Furthermore, we detected the stability of POU6F2-AS1 in SW480 cells upon METTL3 knockdown or overexpression. In cells treated with actinomycin D to block the de novo synthesis of RNA, METTL3 knockdown decreased the stability of POU6F2-AS1, whereas METTL3 overexpression had the opposite effect (Fig. 7J). Next, we elucidated to the mechanism involved in the METTL3-induced stabilization of POU6F2-AS1 in CRC cells. IGF2BP2, a well-known “reader” of m^6^A, is also an RNA binding protein that plays a vital role in controlling the stability of mRNAs [39]. Knockdown of IGF2BP2 in SW480 cells markedly decreased POU6F2-AS1 expression (Fig. 7K). Importantly, knockdown of IGF2BP2 decreased the half-life of POU6F2-AS1 in SW480 cells treated with actinomycin D (Fig. 7L). In addition, the interaction between IGF2BP2 and POU6F2-AS1 was inhibited after METTL3 knockdown (Fig. 7M). Overall, our data indicated that METTL3-mediated m^6^A modification promotes POU6F2-AS1 expression in CRC via IGF2BP2-dependent stabilization of RNA.

## Discussion

Transcriptome studies have demonstrated that protein-coding RNAs account for less than 2% of the transcribed human genome, and most of the remaining RNAs are noncoding RNAs (ncRNAs) [40]. Currently, lncRNAs, which are functional ncRNAs, have attracted increasing amounts of attention in cancer research. Several phase I/II clinical trials targeting lncRNAs have been conducted to treat patients with cancer [41]. The development of aberrantly expressed lncRNAs as therapeutic tools has yielded exciting prospects. Moreover, with the widespread use and improvement of high-throughput sequencing, additional opportunities have been provided for bioinformatics-based research and clinical validation of promising lncRNAs. In the present study, we identified the novel upregulated lncRNA POU6F2-AS1 in CRC via comprehensive analysis of publicly available datasets and validated the upregulation of POU6F2-AS1 in both CRC tissues and cell lines. Moreover, we found that high expression of POU6F2-AS1 was positively associated with advanced clinicopathological stage, large tumour size, and poor OS in patients with CRC, suggesting that POU6F2-AS1 might play an essential role in CRC tumorigenesis and progression. Previous study demonstrated that POU6F2-AS1 are upregulated in lung cancer and CRC and is an oncogenic factor [42, 43]. However, the function and mechanism of POU6F2-AS1 in lipid metabolism has not been explored in CRC.

Gain- and loss-of-function experiments demonstrated that POU6F2-AS1 promotes the growth of CRC cells in vitro and in vivo. In addition, RNA sequencing analysis, BODIPY and Oil Red O staining and LC/MS data revealed that POU6F2-AS1 was able to affect lipid metabolism and FA metabolism. Reprogramming of lipid metabolism is a newly well-documented hallmark of cancer cells, and represents an attractive vulnerability that might be exploited as s therapeutic target [6]. Proliferating cancer cells are likely to obtain energy via lipid reprogramming particularly through the synthesis of FAs; overall, FA metabolism involves FA uptake, de novo synthesis, and β‐oxidation to support unlimited cellular proliferation [19, 20, 44]. In addition, cancer cells can utilize FAs to provide ingredients for synthesizing cellular organelles and membranes during rapid replication [45].

Multiple metabolic pathways contribute to lipid accumulation in cancer cells, and most of these pathways are related to the rate-limiting lipogenic enzymes, whose upregulation induces FA synthesis [46]. Accumulating evidence has shown that multiple lipogenic enzymes, such as FASN, ACC, SCD1, and ACLY, are aberrantly upregulated in a wide variety of cancers, driving FA synthesis and contributing to lipogenesis and the progression of cancer cells [47–50]. Consistently, the expression of lipogenic enzymes is also markedly increased in CRC [51]. Recently, lncRNAs were reported to participate in the regulation of lipogenic enzymes, thereby promoting the progression of cancers. For example, Zheng et al. revealed that the lncRNA TINCR could bind to ACLY and maintain its protein stability by protecting it from ubiquitin-mediated degradation, thereby facilitating de novo lipid biosynthesis and progression of nasopharyngeal carcinoma [52]. Jia et al. reported that that the lncRNA NEAT1 facilitated FA metabolism in gastric cancer via the c‑Jun/c‑Fos/SREBP1 axis [53]. Peng et al. demonstrated that the lncRNA FASRL could bind to ACACA and inhibit its phosphorylation, thus increasing FA synthesis in hepatocellular carcinoma [54]. However, the crosstalk between lncRNAs and metabolic enzymes or processes in CRC is poorly understood. In the present study, our findings revealed that POU6F2-AS1 overexpression induces lipogenesis and proliferation in CRC cells by upregulating FASN, which is the key rate‐limiting lipogenic enzyme responsible for the terminal catalytic step in the synthesis of FAs. FASN is often upregulated in cancer cells and its depletion results in antitumour effects; thus, FASN has been defined as a promising therapeutic target [55]. Consistent with this conclusion, we found that FASN knockdown could efficiently attenuated CRC cell lipogenesis and proliferation, while these processed were promoted by POU6F2-AS1 overexpression. These findings indicated cotreatment with POU6F2-AS1 siRNAs may improve the therapeutic efficacy of FASN inhibitors. However, the antitumour effects of combination therapies based on POU6F2-AS1 need to be further studied in the future.

LncRNAs typically exert their functions by acting as microRNA (miRNA) sponges and interacting with RBPs [56, 57]. In the present study, by overlapping the MS analysis and two online RBP prediction results, we determined that POU6F2-AS1 specifically interacts with YBX1. Deletion-mapping analysis revealed that the 117–195 nt region of POU6F2-AS1 was responsible for the interaction between these two genes, which is consistent with the regions predicted by catRAPID. YBX1 has been shown to play vital roles in the tumorigenesis and progression of malignancies, including CRC. Accumulating evidence has indicated that YBX1 is a vital functional partner of multiple lncRNAs in cancer. For instance, YBX1 is an interactive partner of lincNMR that regulates the expression of downstream RRM2, TYMS and TK1 by binding to their promoters, governing nucleotide metabolism and cell proliferation in cancer [58]. The lncRNA MILIP bound with YBX1 to increase the translation of Snail, leading to the enhanced metastasis of clear cell renal cell carcinoma cells [59]. LncRNA RMRP recruits YBX1 to upregulate the transcription of TGFBR1, thereby promoting the proliferation and progression of non-small cell lung cancer [60]. Previous studies demonstrated that YBX1, a well-established transcription factor, may bind to the promoters of certain genes, thereby initiating their transcriptional activation. For example, NOTCH3 [61], CBX3 [62], and ANXA8 [63] are regulated by YBX1 via transcriptional activation. In this study, we found that POU6F2-AS1 tethers YBX1 to the promoter of FASN to promote FASN activation. Furthermore, ChIP assays revealed that the region spanning from − 583 to -10 nt in the FASN promoter contains the binding sites for YBX1. Coincidently, YBX1 binding site-2 (YBX-2), YBX-3, YBX-5, and YBX-6, which were predicted by the JASPAR website, were also located in this region. These findings are consistent with conclusions reported in the literature that transcriptional regulation is one of the most important mechanisms of FASN overexpression in cancer cells [36]. However, we did not detect whether histone proteins or other transcription factors in this region cooperatively interact with YBX1 to initiate FASN transcription. Additionally, as a multifunctional DNA/RNA binding protein RNA, YBX1 also participates in the regulation of multiple DNA/RNA-dependent events, including pre-mRNA splicing, mRNA packaging, translation and stabilization, etc [29, 64]. However, whether YBX1 cooperates with POU6F2-AS1 to regulate FASN via these mechanisms needs to be explored in the future.

In addition, our work addressed the mechanism of POU6F2-AS1 upregulation in CRC. The m^6^A modification, a type of reversible epigenetic regulatory mechanism, has been widely reported to be involved in the stabilization and expression of RNAs, including lncRNAs, in cancer [65, 66]. For example, METTL3-mediated m^6^A modification increases the stabilization and expression of THAP7-AS1 in an IGF2BP1-dependent manner [37]. METTL3 catalyzes m^6^A modification, and YTHDC1 recognizes and stabilizes m^6^A to increase expression of TERRA [67]. Since the bioinformatics analysis predicted high-confidence m^6^A sites in the sequence of POU6F2-AS1, we further assessed whether POU6F2-AS1 was an m^6^A-incucible lncRNA. Coincidently, we observed that knockdown of METTL3 suppressed the expression and stability of POU6F2-AS1 in CRC cells and decreased m^6^A level of POU6F2-AS1. IGF2BP2 has been shown to mediate the stability of m^6^A-modified RNAs, such as CREB1 [68], HMGA1 [69], MSX1 and JARID2 [70] in CRC. Here, we also found that IGF2BP2 could recognize m^6^A sites and maintain the stability of POU6F2-AS1. Overall, these results suggested that METTL3-catalyzed and IGF2BP2- stabilized m^6^A modification may be involved in the upregulation of POU6F2-AS1 in CRC.

## Conclusions

In summary, POU6F2-AS1 is a novel upregulated lncRNA in CRC that is stabilized by METTL3-mediated m^6^A modification. Moreover, POU6F2-AS1 specifically tethers YBX1 to the FASN promoter to activate its transcription, leading to the enhanced expression of this key enzyme in the de novo synthesis of FAs, thereby facilitating the progression of CRC. Thus, POU6F2-AS1 plays a pivotal role in the oncogenic METTL3/POU6F2-AS1/YBX1/FASN axis to promote CRC cell lipogenesis and growth, which provides new insight into lipid accumulation in CRC and highlights, POU6F2-AS1 as a promising therapeutic target for patients with CRC.

### Electronic supplementary material

Below is the link to the electronic supplementary material.

**Supplementary Material 1**: Original western blot images

**Supplementary Material 2**: **Fig. S1**. (A-D) Volcano plots of the differentially expressed genes for GSE126092, GSE134525, GSE109454, and GSE84983. (E) Analysis of POU6F2-AS1 expression in cell lines of different tissue origins in the Cancer Cell Line Encyclopedia (CCLE) database. (F) FISH staining intensities of POU6F2-AS1 in CRC tissues compared with paired ANTs. T, tumour tissues; N, paired adjacent non-cancerous tissues. (G) The FISH score of POU6F2-AS1 in CRC tissues was remarkably higher than that in paired ANTs. (H, I) Validation of POU6F2-AS1 expression in HCT116 and SW480 cells transfected with siRNAs. (J) Validation of POU6F2-AS1 expression in LoVo cells transfected with overexpression plasmids. (K) Gene Ontology (GO) analysis including biological process (BP), cellular component (CC) and molecular function (MF) exhibited the significantly enriched pathways after POU6F2-AS1 overexpression. *P < 0.05, **P < 0.01, ***P < 0.001

**Supplementary Material 3**: **Fig. S2**. (A-C) Relative triglyceride levels in CRC cells with POU6F2-AS1 knockdown or overexpression. (D-H) CCK–8, EdU and colony formation analysis of PA in HCT116 and SW480 cells with POU6F2-AS1 knockdown. (I-K) CCK–8, EdU and colony formation analysis of orlistat in LoVo cells with POU6F2-AS1 overexpression. The data are presented as the mean ± SD. *P < 0.05, **P < 0.01, ***P < 0.001.

**Supplementary Material 4**: **Fig. S3**. (A) Representative RNA-FISH images showing that the localization of POU6F2-AS1 in CRC cells. POU6F2-AS1 probes are red, and nuclei are stained with DAPI. (B) Correlation of POU6F2-AS1 and YBX1 expression in CRC was analyzed by GEPIA 2 website. (C) The secondary structure of POU6F2-AS1 predicted by RNA fold website. (D) The YBX1 expression between CRC tissues and normal samples in TCGA database. (E) The YBX1 expression between CRC tissues and paired normal samples in TCGA database. (F, G) Validation of YBX1 expression by qRT–PCR and western blotting in LoVo cells transfected with siRNAs. (H) CRC patient-derived organoid infected with POU6F2-AS1 knockdown or YBX1 overexpression lentivirus. (I) Colony formation assay in POU6F2-AS1 knockdown of HCT116 cells with YBX1 overexpression. (J) Oil Red O staining assay in POU6F2-AS1 knockdown of HCT116 cells with YBX1 overexpression. The data are presented as the mean ± SD. *P < 0.05, **P < 0.01, ***P < 0.001.

**Supplementary Material 5**: **Fig. S4**. (A, B) Correlation of FASN and POU6F2-AS1 or YBX1 expression in CRC was analyzed by GEPIA 2 website. (C) Luciferase reporter assays showed that the luciferase activity driven by the -447/-136 fragment of the FASN promoter region was significantly reduced in SW480 cells with YBX1 knockdown. (D, E) The FASN expression between CRC tissues and normal samples in TCGA database. (F, G) Validation of FASN expression by qRT–PCR and western blotting in LoVo cells transfected with siRNAs. (H) Validation of FASN expression by qRT–PCR in HCT116 cells transfected with FASN overexpression plasmid. (I-K) CCK–8, colony formation and Oil Red O staining assays with POU6F2-AS1 knockdown in SW480 cells with FASN overexpression. (L-N) Colony formation, CCK–8 and Oil Red O staining assays in POU6F2-AS1 overexpression LoVo cells with FASN knockdown. (O, P) Western blotting validation of METTL3 protein expression after transfection of shRNAs and overexpression plasmids into CRC cells, respectively. (Q) After transfecting overexpression plasmid of METTL3 into SW480 cells, the mRNA expression of METTL3 and POU6F2-AS1 was verified by qRT–PCR. The data are presented as the mean ± SD. *P < 0.05, **P < 0.01, ***P < 0.001

**Supplementary Material 6**: **Additional file 1: Table S4**. The sequences of primers used for qRT–PCR

**Supplementary Material 7**: **Additional file 1: Table S1**. Relationship between POU6F2-AS1 expression and clinicopathological features in TMAs cohort

**Supplementary Material 8**: **Additional file 1: Table S2**. Proteins identified by mass spectrometry

**Supplementary Material 9**: **Additional file 1: Table S3**. The sequences of oligonucleotides and probes used in this study

**Supplementary Material 10**: **Additional file 2**: Supplemental Materials and Methods

## Data Availability

The RAN-seq datasets of dysregulated lncRNAs in CRC were obtained from GEO database (https://www.ncbi.nlm.nih.gov/geo/) under the correspond accession number. The public available datasets involved in this study can be obtained from online repositories. All datasets involved in the present study are available from the corresponding author on reasonable request

## Abbreviations

CRC: Colorectal cancer
TCGA: The Cancer Genome Atlas
LncRNA: long non-coding RNA
FA: Fatty acid
YBX1: Y-box binding protein 1
FASN: Fatty Acid Synthase
ROC: Receiver operating characteristic
HE: Hematoxylin eosin
OS: overall survival
KEGG: Kyoto encyclopedia of genes and genomes
shRNA: short hairpin RNA
siRNA: small interfering RNA
GEPIA: The Gene Expression Profiling Interactive Analysis
ANTs: adjacent noncancerous tissues
qRT–PCR: Real-time quantitative polymerase chain reaction
CCK–8: Cell Counting Kit–8
FISH: RNA fluorescence in situ hybridization
IHC: Immunohistochemical staining
Edu: 5-Ethynyl-2’-deoxyuridine
TAG: Triglyceride
RIP: RNA immunoprecipitation
Me-RIP: Methylated RNA Immunoprecipitation
ChIP: Chromatin immunoprecipitation
PDO: Patient-derived organoid
GEO: Gene-Expression Omnibus
LMGs: Lipid metabolism-related genes
PA: Palmitic acid
LC-MS: Liquid chromatography mass spectrometry
