# Supplementary material for: The m6A modification mediated-lncRNA POU6F2-AS1 reprograms fatty acid metabolism and facilitates the growth of colorectal cancer via upregulation of FASN
Source: Mol Cancer. 2024 Mar 16;23:55. doi: 10.1186/s12943-024-01962-8 (PMC10943897; doi:10.1186/s12943-024-01962-8)
Supplement: Supplementary file 1 — Supplementary Material 1: Original western blot images [file 12943_2024_1962_MOESM1_ESM.docx]

**Fig.3L**







FASN

FASN

SW480

HCT116







ACC1

ACC1

SW480

HCT116







SW480

HCT116

SCD1

SCD1







CD36

CD36

SW480

HCT116







CPT1A

CPT1A

SW480

HCT116







β-actin

β-actin

SW480

HCT116

**Fig.3N**







ACC1

FASN







CD36

SCD1







β-actin

CPT1A

**Fig.4D**


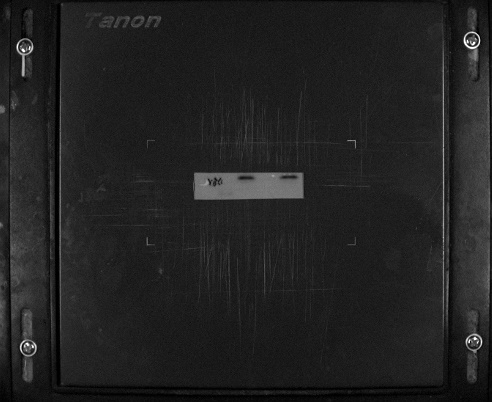


YBX1

**Fig.4I**


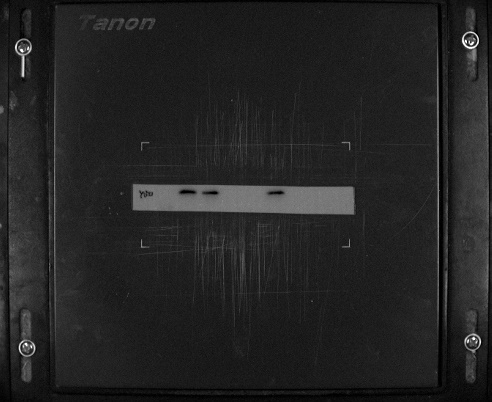


YBX1

**Fig.4K**


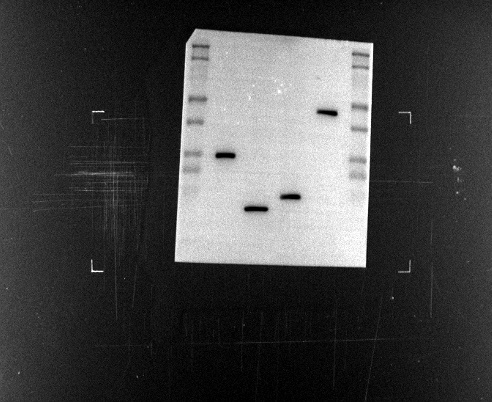

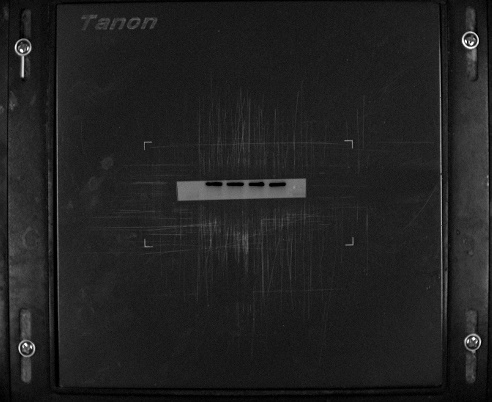


β-actin

Flag

**Fig.5B**


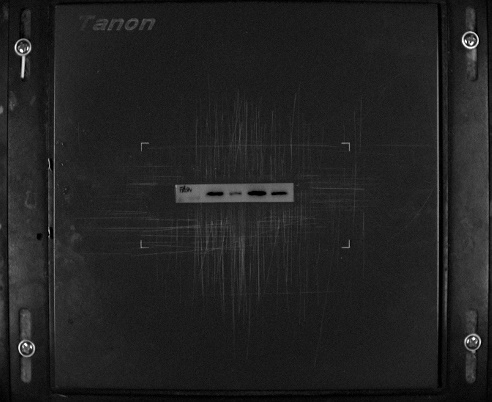

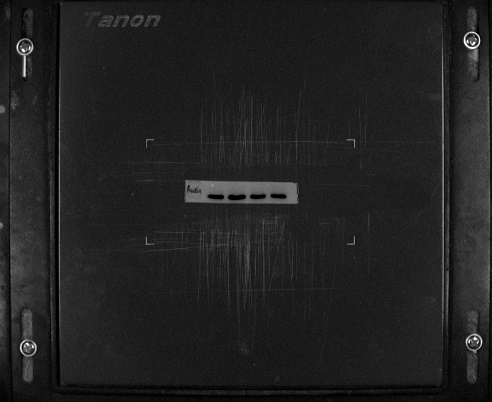


HCT116

β-actin

FASN

HCT116


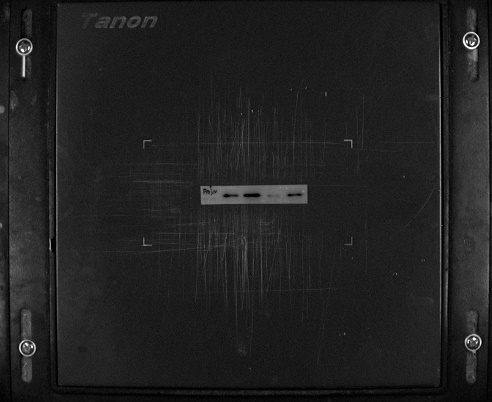

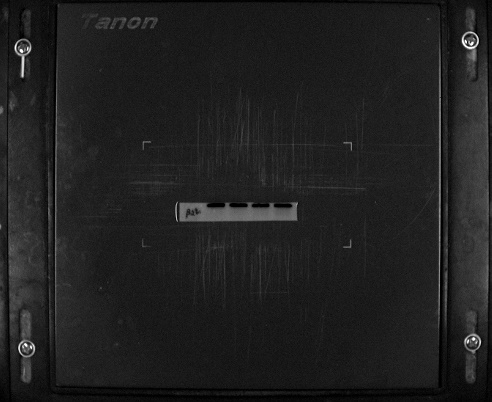


SW480

SW480

β-actin

FASN

**Fig.S3G**







β-actin

YBX1

**Fig.S4G**







FASN

β-actin

**Fig.S4O**


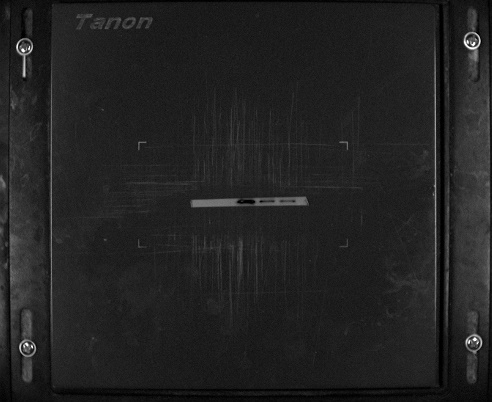

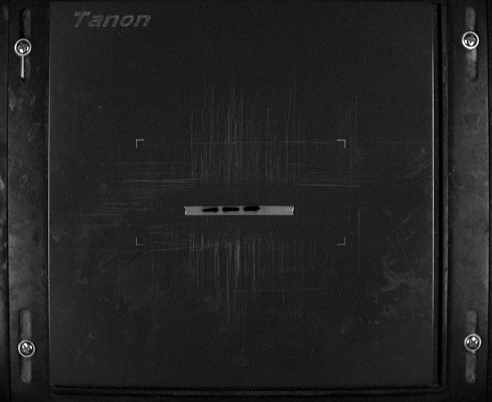


β-actin

METTL3

**Fig.S4P**







METTL3

β-actin
