# Supplementary figures and images for: The m6A modification mediated-lncRNA POU6F2-AS1 reprograms fatty acid metabolism and facilitates the growth of colorectal cancer via upregulation of FASN
Source: Mol Cancer. 2024 Mar 16;23:55. doi: 10.1186/s12943-024-01962-8 (PMC10943897; doi:10.1186/s12943-024-01962-8)

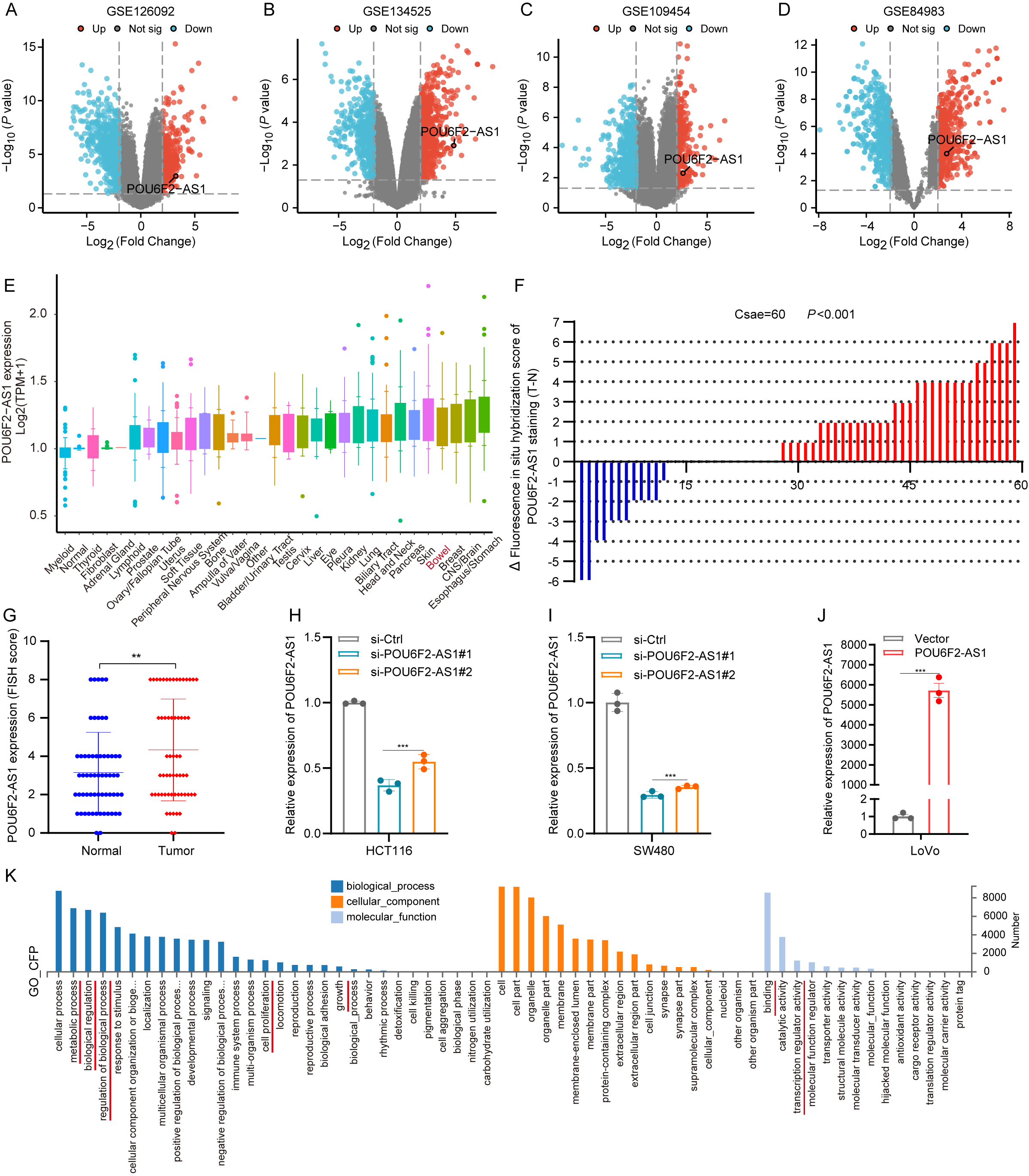

Supplement: Supplementary file 2 — Supplementary Material 2: Fig. S1. (A-D) Volcano plots of the differentially expressed genes for GSE126092, GSE134525, GSE109454, and GSE84983. (E) Analysis of POU6F2-AS1 expression in cell lines of different tissue origins in the Cancer Cell Line Encyclopedia (CCLE) database. (F) FISH staining intensities of POU6F2-AS1 in CRC tissues compared with paired ANTs. T, tumour tissues; N, paired adjacent non-cancerous tissues. (G) The FISH score of POU6F2-AS1 in CRC tissues was remarkably higher than that in paired ANTs. (H, I) Validation of POU6F2-AS1 expression in HCT116 and SW480 cells transfected with siRNAs. (J) Validation of POU6F2-AS1 expression in LoVo cells transfected with overexpression plasmids. (K) Gene Ontology (GO) analysis including biological process (BP), cellular component (CC) and molecular function (MF) exhibited the significantly enriched pathways after POU6F2-AS1 overexpression. *P < 0.05, **P < 0.01, ***P < 0.001 [file 12943_2024_1962_MOESM2_ESM.tif]

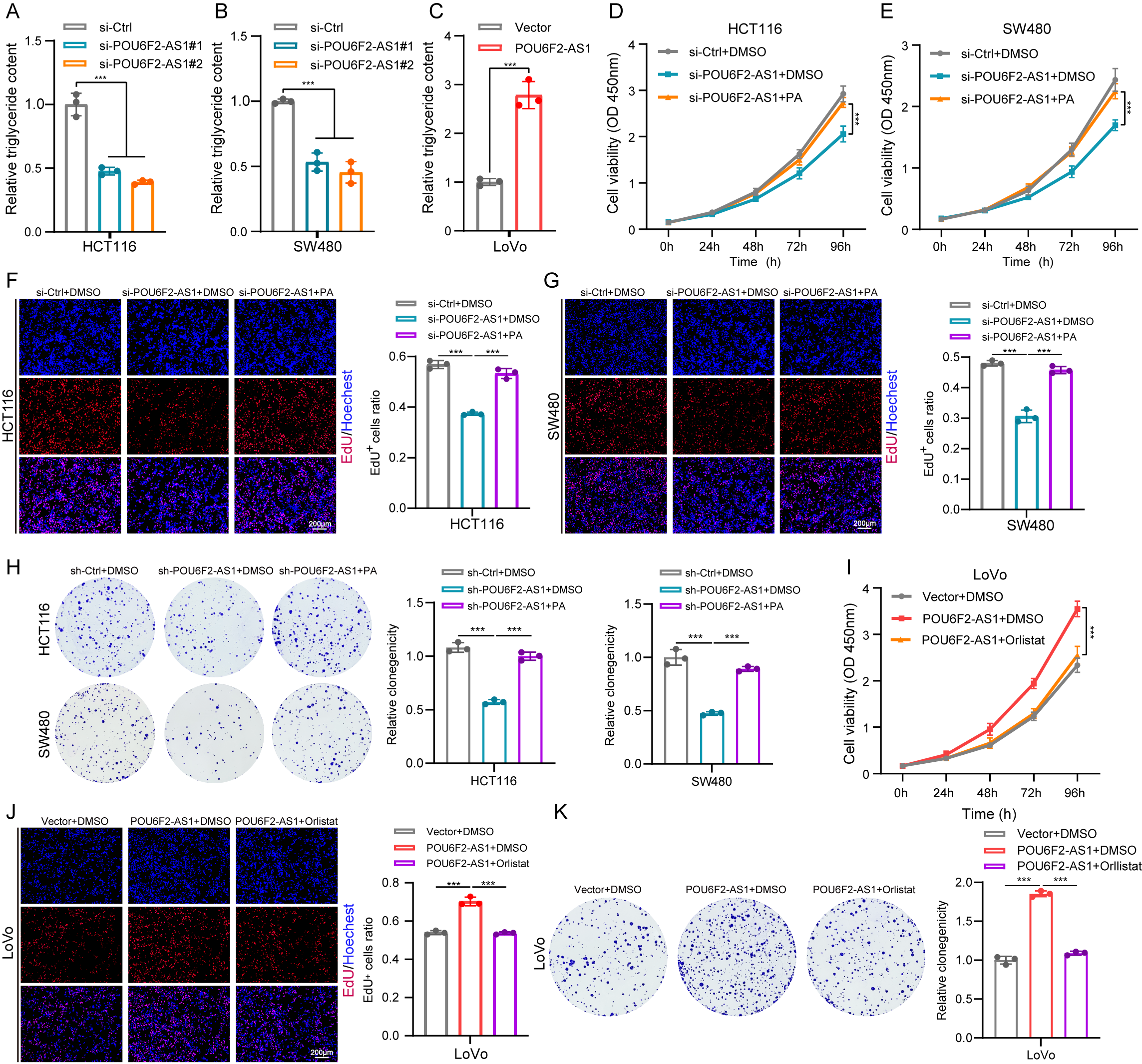

Supplement: Supplementary file 3 — Supplementary Material 3: Fig. S2. (A-C) Relative triglyceride levels in CRC cells with POU6F2-AS1 knockdown or overexpression. (D-H) CCK–8, EdU and colony formation analysis of PA in HCT116 and SW480 cells with POU6F2-AS1 knockdown. (I-K) CCK–8, EdU and colony formation analysis of orlistat in LoVo cells with POU6F2-AS1 overexpression. The data are presented as the mean ± SD. *P < 0.05, **P < 0.01, ***P < 0.001. [file 12943_2024_1962_MOESM3_ESM.tif]

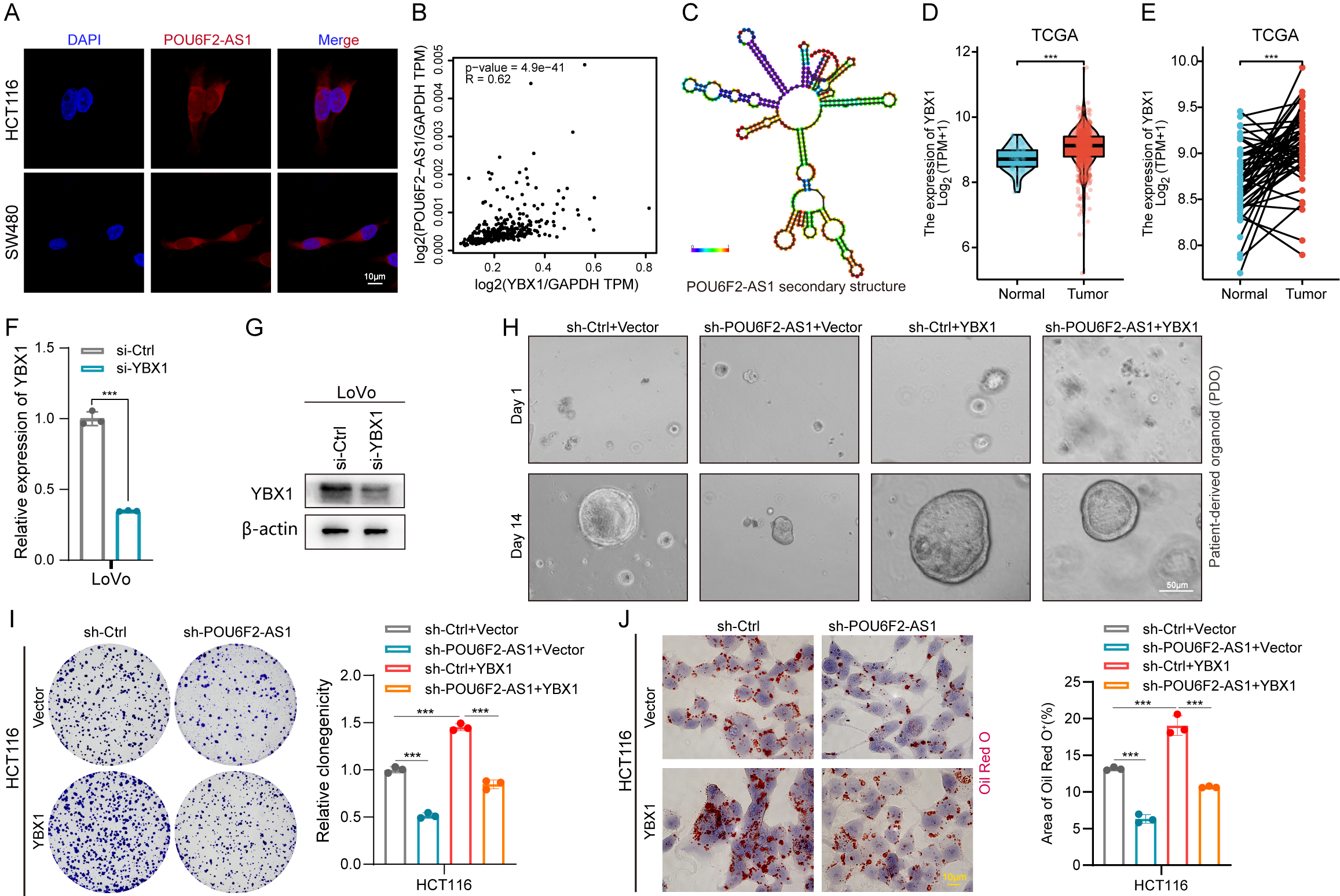

Supplement: Supplementary file 4 — Supplementary Material 4: Fig. S3. (A) Representative RNA-FISH images showing that the localization of POU6F2-AS1 in CRC cells. POU6F2-AS1 probes are red, and nuclei are stained with DAPI. (B) Correlation of POU6F2-AS1 and YBX1 expression in CRC was analyzed by GEPIA 2 website. (C) The secondary structure of POU6F2-AS1 predicted by RNA fold website. (D) The YBX1 expression between CRC tissues and normal samples in TCGA database. (E) The YBX1 expression between CRC tissues and paired normal samples in TCGA database. (F, G) Validation of YBX1 expression by qRT–PCR and western blotting in LoVo cells transfected with siRNAs. (H) CRC patient-derived organoid infected with POU6F2-AS1 knockdown or YBX1 overexpression lentivirus. (I) Colony formation assay in POU6F2-AS1 knockdown of HCT116 cells with YBX1 overexpression. (J) Oil Red O staining assay in POU6F2-AS1 knockdown of HCT116 cells with YBX1 overexpression. The data are presented as the mean ± SD. *P < 0.05, **P < 0.01, ***P < 0.001. [file 12943_2024_1962_MOESM4_ESM.tif]

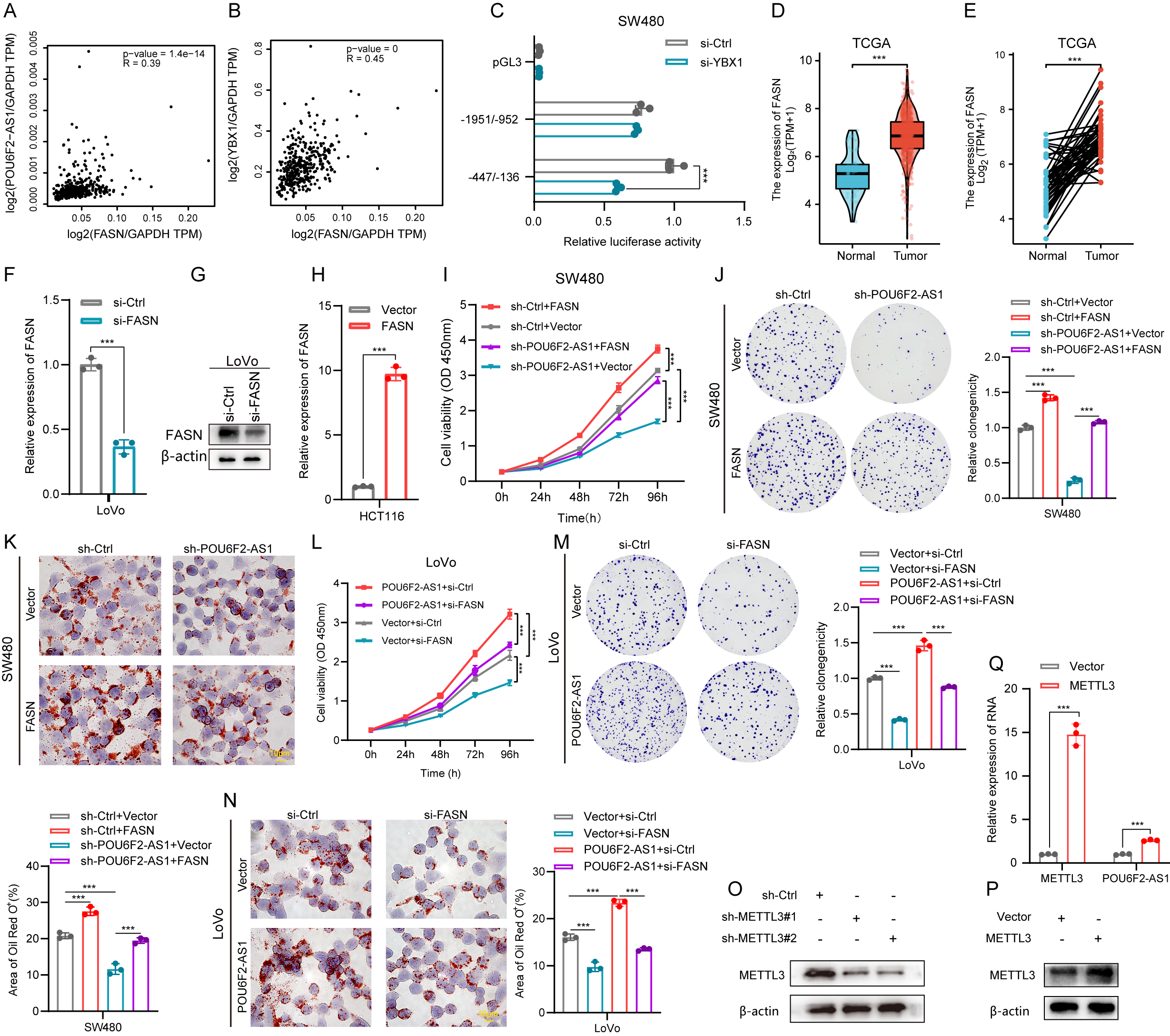

Supplement: Supplementary file 5 — Supplementary Material 5: Fig. S4. (A, B) Correlation of FASN and POU6F2-AS1 or YBX1 expression in CRC was analyzed by GEPIA 2 website. (C) Luciferase reporter assays showed that the luciferase activity driven by the -447/-136 fragment of the FASN promoter region was significantly reduced in SW480 cells with YBX1 knockdown. (D, E) The FASN expression between CRC tissues and normal samples in TCGA database. (F, G) Validation of FASN expression by qRT–PCR and western blotting in LoVo cells transfected with siRNAs. (H) Validation of FASN expression by qRT–PCR in HCT116 cells transfected with FASN overexpression plasmid. (I-K) CCK–8, colony formation and Oil Red O staining assays with POU6F2-AS1 knockdown in SW480 cells with FASN overexpression. (L-N) Colony formation, CCK–8 and Oil Red O staining assays in POU6F2-AS1 overexpression LoVo cells with FASN knockdown. (O, P) Western blotting validation of METTL3 protein expression after transfection of shRNAs and overexpression plasmids into CRC cells, respectively. (Q) After transfecting overexpression plasmid of METTL3 into SW480 cells, the mRNA expression of METTL3 and POU6F2-AS1 was verified by qRT–PCR. The data are presented as the mean ± SD. *P < 0.05, **P < 0.01, ***P < 0.001 [file 12943_2024_1962_MOESM5_ESM.tif]
