# Supplementary material for: The m6A modification mediated-lncRNA POU6F2-AS1 reprograms fatty acid metabolism and facilitates the growth of colorectal cancer via upregulation of FASN
Source: Mol Cancer. 2024 Mar 16;23:55. doi: 10.1186/s12943-024-01962-8 (PMC10943897; doi:10.1186/s12943-024-01962-8)
Supplement: Supplementary file 6 — Supplementary Material 6: Additional file 1: Table S4. The sequences of primers used for qRT–PCR [file 12943_2024_1962_MOESM6_ESM.docx]

**Table S4.** The sequences of primers used for qRT-PCR.

| **Primer names** | **Primer sequences** |
| --- | --- |
| POU6F2-AS1 | Forward: ATTTATGGCCTCCGTGCTCTC |
|  | Reverse: CCCATGGTGGTAATCTGGGA |
| METTL3 | Forward: ATCCCCAAGGCTTCAACCAG |
|  | Reverse: GCGAGTGCCAGGAGATAGTC |
| FASN | Forward: AGTACACACCCAAGGCCAAG |
|  | Reverse: GGATACTTTCCCGTCGCATA |
| YBX1 | Forward: CCAGGAAGTACCTTCGCAGTG |
|  | Reverse: AGGACCCCTACGACGTGGAT |
| IGF2BP2 | Forward: GTTGGTGCCATCATCGGAAAGG |
|  | Reverse: TGGATGGTGACAGGCTTCTCTG |
| ACC1 | Forward: TCACACCTGAAGACCTTAAAGCC |
|  | Reverse: AGCCCACACTGCTTGTACTG |
| SCD1 | Forward: GTACCGCTGGCACATCAACTT |
|  | Reverse: TTGGAGACTTTCTTCCGGTCAT |
| CD36 | Forward: CTTTGGCTTAATGAGACTGGGAC |
|  | Reverse: GCAACAAACATCACCACACCA |
| CPT1A | Forward: GATCCTGGACAATACCTCGGAG |
|  | Reverse: CTCCACAGCATCAAGAGACTGC |
| P1 | Forward: AGAGGGAGCCAGAGAGACG |
|  | Reverse: GGCTGCTCGTACCTGGTGA |
| P2 | Forward: GCACGAGCATCACCCCA |
|  | Reverse: CGGCGCCGACGCTATTTA |
| P3 | Forward: ATATAACACGGGGGTCGGGGAT |
|  | Reverse: TTTAATGGTGGGGCTGGGACTGA |
| P4 | Forward: GATTGGTTGCTGCTGCCG |
|  | Reverse: TGTGCGGGATGGGAATGCT |
| P5 | Forward: CCGGGAAGCTGCTAAGGAG |
|  | Reverse: GTTGCTAGGCGATAGGGTGA |
| P6 | Forward: AAGCGCGAAGCGGTCAGAA |
|  | Reverse: TGGTGTGGTGCGGACAAAGG |
| P7 | Forward: CTTCCCTTGTCCTTCCTTGACC |
|  | Reverse: TTGTGAGGCCACAGGTGTCT |
| P8 | Forward: CGAGTCTCCAACCTCCTTGG |
|  | Reverse: CTGCGGGGGTTCTTCTGATT |
| P9 | Forward: GTAGCTGGGACTAGAGGCA |
|  | Reverse: GCTTCCACAGAAAGTGAGCG |
| GAPDH | Forward: AAGGTCGGAGTCAACGGATTTG |
|  | Reverse: CCATGGGTGGAATCATATTGGAA |
