# Supplementary material for: The m6A modification mediated-lncRNA POU6F2-AS1 reprograms fatty acid metabolism and facilitates the growth of colorectal cancer via upregulation of FASN
Source: Mol Cancer. 2024 Mar 16;23:55. doi: 10.1186/s12943-024-01962-8 (PMC10943897; doi:10.1186/s12943-024-01962-8)
Supplement: Supplementary file 7 — Supplementary Material 7: Additional file 1: Table S1. Relationship between POU6F2-AS1 expression and clinicopathological features in TMAs cohort [file 12943_2024_1962_MOESM7_ESM.docx]

**Table S1** Relationship between POU6F2-AS1 expression and clinicopathological features in CRC patients.

| **Variables** | **Cases** | **POU6F2-AS1 expression (n=60)** | | ***P* ^a^** |
| --- | --- | --- | --- | --- |
|  |  | **Low (n=30)** | **High (n=30)** |  |
| Age (years) |  |  | | 0.067 |
| ≤60 | 25 | 9 | 16 |  |
| >60 | 35 | 21 | 14 |  |
| Gender |  |  | | 0.292 |
| Female | 24 | 10 | 14 |  |
| Male | 36 | 20 | 16 |  |
| Tumor diameter (cm) |  |  | | 0.038 |
| ≤5 | 32 | 20 | 12 |  |
| >5 | 28 | 10 | 18 |  |
| Depth of invasion |  |  | | <0.001 |
| T1-T2 | 29 | 21 | 8 |  |
| T3-T4 | 31 | 9 | 22 |  |
| Lymph node metastasis |  |  | | 0.010 |
| N0 | 28 | 19 | 9 |  |
| N1/N2 | 32 | 11 | 21 |  |
| Distant metastasis |  |  | | 0.612 |
| M0 | 56 | 29 | 27 |  |
| M1 | 4 | 1 | 3 |  |
| TNM stage |  |  | | 0.010 |
| I-II | 28 | 19 | 9 |  |
| III-IV | 32 | 11 | 21 |  |

*P*-value ^a^ was measured by Pearson’s Chi-Squared or Fisher’s exact test.
