# Supplementary material for: The m6A modification mediated-lncRNA POU6F2-AS1 reprograms fatty acid metabolism and facilitates the growth of colorectal cancer via upregulation of FASN
Source: Mol Cancer. 2024 Mar 16;23:55. doi: 10.1186/s12943-024-01962-8 (PMC10943897; doi:10.1186/s12943-024-01962-8)
Supplement: Supplementary file 9 — Supplementary Material 9: Additional file 1: Table S3. The sequences of oligonucleotides and probes used in this study [file 12943_2024_1962_MOESM9_ESM.docx]

| **siRNAs** | |
| --- | --- |
| si-Ctrl sense | UUCUCCGAACGUGUCACGUTT |
| si-POU6F2-AS1#1 sense | GCCUAAAGAGAAACCCUGUTT |
| si-POU6F2-AS1#2 sense | GGUACAGACACUGCCCAUUTT |
| si-YBX1 sense | CCACGCAAUUACCAGCAAATT |
| si-FASN sense | GCUACGACUACGGCCCUCATT |
| si-IGF2BP2 sense | CAGUUUGAGAACUACUCCUTT |
| **shRNAs** |  |
| sh-Ctrl sense | TTCTCCGAACGTGTCACGT |
| sh-POU6F2-AS1 sense | GCCTAAAGAGAAACCCTGT |
| sh-METTL3#1 sense | AAGTATGTTCACTATGAAA |
| sh-METTL3#2 sense | CAAGGAACAATCCATTGTT |

**Table S3.** The sequences of oligonucleotides and probes used in this study.
