# Supplementary material for: The m6A modification mediated-lncRNA POU6F2-AS1 reprograms fatty acid metabolism and facilitates the growth of colorectal cancer via upregulation of FASN
Source: Mol Cancer. 2024 Mar 16;23:55. doi: 10.1186/s12943-024-01962-8 (PMC10943897; doi:10.1186/s12943-024-01962-8)
Supplement: Supplementary file 10 — Supplementary Material 10: Additional file 2: Supplemental Materials and Methods [file 12943_2024_1962_MOESM10_ESM.docx]

**Materials and methods**

**Cell transfection**

siRNAs targeting POU6F2-AS1 (si-POU6F2-AS1#1, si-POU6F2-AS1#2), YBX1 (si-YBX1), FASN (si-FASN), and IGF2BP2 (si-IGF2BP2) and si-Ctrl were designed and synthesized by Gene Pharma Technology (Shanghai, China). The CRC cells were transfected with the siRNAs by jetPRIME^®^ Polyplus Transfection (Polyplus-transfection S.A., Illkirch, France) according to the to the manufacturer’s instructions. To construct overexpression plasmids, the full-length sequences of POU6F2-AS1, YBX1, FASN and METTL3 were cloned into the pcDNA3.1 vector (Invitrogen, Shanghai, China), while empty vector was used as a negative control. CRC cells were transfected with these plasmids by Hieff Trans™ Liposomal Transfection Reagent (Yeasen Biotechnology, Shanghai, China) according to the manufacturer’s instructions. The sequences of the siRNA and shRNA are described in the Additional file 1: Table S3

**RNA extraction and qRT–PCR**

Total RNA was isolated from CRC specimens and cells using RNA Isolater Total RNA Extraction Reagent (Vazyme, Nanjing, China) according to the manufacturer’s guidelines. The quality and concentration of the extracted samples were determined by a Nanodrop 2000 (Thermo Fisher Scientific, USA). For qRT–PCR analysis of POU6F2-AS1 and mRNA expression, RNA was first reverse-transcribed to cDNA using HiScript II Q RT SuperMix (Vazyme, Nanjing, China), which was then detected by ChamQ SYBR qPCR Master Mix (Vazyme, Nanjing, China) on a LightCycler 96 Instrument (Roche, Switzerland) using the following thermal conditions: 95 °C for 30 s; 40 cycles at 95 °C for 10 s and 60 °C for 60 s; and a melting curve analysis. The relative RNA expression level was calculated using the 2^-ΔΔCT^ method, GAPDH serving as a reference for RNAs. The sequences of the primers used are listed in Additional file 1: Table S4.

**Immunohistochemistry (IHC)**

The collected subcutaneous tumors were fixed with 4% formalin, embedded in paraffin and then sectioned into 4-μm-slices. The standard protocol for IHC of the TMAs and subcutaneous tumors using a streptavidin-peroxidase (SP) Kit (Zhongshan biotech, Beijing, China) according to the manufacturer’s instructions. The slides were incubated with antibodies specific for FASN (1:100, Proteintech, IL, USA), METTL3 (1:500, Proteintech, IL, USA) and Ki-67 (1:2500, Proteintech, IL, USA). The images of IHC staining were obtained by an Olympus microscope (Tokyo, Japan). Three pathologists assessed separately the TMAs under blinded experimental conditions and all differences that arise were resolved by discussion. The staining score was evaluated by a combination of the intensity of the immunostaining and the percentage of immunoreactive cells and was quantified by immunoreactivity score (IRS). The intensity of immunostaining was scored as 0–3 (0, negative; 1, weak; 2, moderate; 3, strong); the percentage of immunoreactivity cells was graded as 1 (0–25%), 2 (26–50%), 3 (51–75%), and 4 (76–100%). The IRS was obtained by multiplying the intensity of immunostaining and the percentage of immunoreactive cells. Relied on the IRS, the level of METTL3 expression was categorized as low (IRS: 0–6) and high (IRS: 8–12) expression.

**RNA stability assay**

For the RNA stability assay, actinomycin D (GIPBIO, CA, USA) was used to inhibit transcription. Briefly, CRC cells with or without METTL3/IGF2BP2 knockdown or overexpression were treated with actinomycin D with a final concentration of 2 μg/ml to terminate transcription and were then collected at 0 h, 4 h, 8 h, 12 h and 16 h, respectively. RNA was extracted from the cells and analyzed via qRT‐PCR.

**Fluorescence in situ hybridization (FISH) and immunofluorescence (IF) staining**

POU6F2-AS1 probe was designed and synthesized by RIBOBIO (Guangzhou, China). The probes were used to detect the location of POU6F2-AS1 in CRC cells by a FISH Kit (RIBOBIO, Guangzhou, China)) according to the manufacturer’s instructions. Cells for IF grown on glass coverslips were fixed with 4% paraformaldehyde (PFA, Sigma Aldrich), permeabilized with 0.2% Triton X-100, blocked with BSA and incubated with anti-YBX1 antibody (1:200, Proteintech, IL, USA) at 4°C overnight. Then incubated for 1 h with the secondary antibody CoraLite488-conjugated goat anti-rabbit IgG (H + L) (1:200, Proteintech, IL, USA) in 37 °C. Besides, 4′,6-diamidino-2-phenylindole, dihydrochloride (DAPI) was used to stain the nuclei of CRC cells. Finally, representative images were taken by an confocal laser scanning microscope (CLSM, Leica STELLARIS 5, Germany). In addition, the expression level of POU6F2-AS1 in TMAs was evaluated by FISH staining and he intensity of POU6F2-AS1 staining was scored as follows: 0, no staining; 1, low staining; and 2, high staining. The percentage of positively stained cells was scored as follows: 0, 0% (no stained cells); 1, 1 ~ 24%; 2, 25 ~ 49%; 3, 50 ~ 74%; and 4, 75 ~ 100%. By multiplying the scores for the staining intensity and the percentage of positively stained cells, the final score was calculated. Then, the CRC samples in TMAs were divided into ow expression group (score 0–3) and the high expression group (score 4–8).

**Western blot**

The transfected CRC cells were harvested and lysed with RIPA buffer (Beyotime, Shanghai, China) supplemented with protease inhibitor cooktail (Thermo Fisher Scientific, USA) and PMSF and quantified by a BCA Protein Assay kit (Beyotime, Shanghai, China). Then, the prepared protein lysates were loaded into a 10% SDS-PAGE gel, transferred onto PVDF membranes (Millipore, MA, USA). After blocked with 5% skimmed milk, the PVDF membranes were incubated with primary antibodies specific against: FASN (1:5000, Proteintech, IL, USA), METTL3 (1:1000, Proteintech, IL, USA), YBX1 (1:5000, Proteintech, IL, USA), CPT1A (1:10000, Proteintech, IL, USA), CD36 (1:5000, Proteintech, IL, USA), SCD (1:5000, Proteintech, IL, USA), ACC1 (1:1000, Cell Signaling Technology, MA, USA), β-actin (1:10000, Proteintech, IL, USA), Flag (1:10000, Proteintech, IL, USA) at 4℃ overnight. The day after, the PVDF membranes were incubated with corresponding secondary antibody for 2 hours at room temperature before washed with TBST buffer for three times. Finally, Chemistar™ High-sig ECL Western Blot Substrate (Tanon, Shanghai, China) was used to detected the blots.

**Cell Counting Kit-8 (CCK-8) assay**

The CCK-8 assay was performed by Cell Counting Kit 8 (GIPBIO, CA, USA) according to the manufacturer’s instructions. Transfected CRC cells were seed in 96-well plates and cultured at 37°C in 5 % CO_2_ for 0 h, 24 h, 48 h, 72 h and 96 h. At specified time‑points, the cells in each well will be treated with 10μL CCK-8 solution and cultured for another 2 hours. Subsequently, the absorbance was measured at 450 nm at the specified time‑points. All the experiments were performed in triplicate.

**5-Ethynyl-2’-deoxyuridine (EdU) assay**

The EdU assay was performed by an Edu Proliferation Kit (RIBOBIO, Guangzhou, China) according to the manufacturer’s instructions. Transfected CRC cells were seed in 96-well plates and cultured at 37°C in 5 % CO_2_ for 12 h, after which the cells were treated with 50 mM EdU solution at 37°C in 5 % CO_2_ for 2 h and then fixed in 4% paraformaldehyde. Subsequently, the cells were stained with Apollo Dye Solution and Hoechst 33342, the photographed and counted by Olympus FSX100 microscope (Olympus, Tokyo, Japan). These experiments were performed in triplicate.

**Colony formation assay**

Transfected CRC cells were seed in 35mm cell culture dishes (800 cells/well) and cultured in corresponding medium supplemented with 10% FBS for two weeks. Subsequently, the cells were fixed with 4% paraformaldehyde solution for 30 minutes before washed by PBS, and then stained with 0.3% crystal violet for another 15 minutes. Finally, the number of colonies was counted and analyzed.

**Methylated RNA Immunoprecipitation (MeRIP)-qPCR**

MeRIP was performed using a riboMeRIP m^6^A Transcriptome Profiling Kit (RIBOBIO, China) according to the manufacturer’s protocol. Total RNA was extracted from cells using Trizol (Vazyme, Nanjing, China). Magnetic beads A/G were prewashed and incubated with 5 μg of anti-m^6^A antibody (Proteintech, IL, USA) or normal rabbit IgG (Proteintech, IL, USA) for 30min at room temperature with rotation. Subsequently, the antibody-conjugated beads were mixed with fragmented RNA, and IP buffer supplemented with RNase inhibitor. Finally, m^6^A bound RNA was extracted with Trizol and the RNA level of POU6F2-AS1 was measured by qRT-PCR.

**RNA immunoprecipitation (RIP)-qPCR**

RIP was performed with a Magna RNA-Binding Protein Immunoprecipitation Kit (Millipore, MA, USA) according to the manufacturer’s instructions. Briefly, transfected cells were lysed on ice for 15 min with RIP Lysis Buffer containing protease inhibitor cocktail and RNase inhibitor, and then centrifuged to collect the supernatant. The beads-antibody complex was obtained by incubating 5 μg of anti-IGF2BP2 antibody (Proteintech, IL, USA), anti-YBX1 antibody (Proteintech, IL, USA), or normal rabbit IgG (Proteintech, IL, USA) and magnetic beads protein A/G with rotation for 30 min at room temperature. Next, the cell lysate supernatant and the complex prepared above were rotationally incubated at 4°C overnight. Each immunoprecipitant flag was resuspended in proteinase K buffer and incubated at 55 °C for 30 min. Finally, RNA was extracted by phenol, chloroform and isoamyl alcohol according to the manufacturer’s instructions, and was detected by qRT-PCR.

**RNA Sequencing**

Total RNA was extracted from vector and overexpression of POU6F2-AS1 CRC cells using Trizol reagent (Vazyme, Nanjing, China). The quantity and quality of RNA were measured by a Nanodrop 2000 (Thermo Fisher Scientific, USA). Library construction and transcriptome sequencing were performed by BGI Genomics (Shenzhen, China). For gene expression analysis, the significance of the differential expression genes was defined according to the combination of the absolute value of |log FC|≥1 and *P* value < 0.05.

**Bioinformatics analysis**

GSE126092, GSE134525, GSE109454, and GSE84983 were downloaded from the Gene-Expression Omnibus (GEO) database (https://www.ncbi.nlm.nih.gov/geo/) to discover dysregulated lncRNAs in CRC. The expression of POU6F2-AS1 in cancers or specific cancer subtypes and normal tissues, as well as the corresponding CRC patients' clinical stage, and ROC curves were obtained from the [Cancer Genome Atlas](https://www.genome.gov/Funded-Programs-Projects/Cancer-Genome-Atlas) (TCGA) database (<https://portal.gdc.cancer.gov/>). The correlation between different genes was detected in the GEPIA 2 website (http://gepia2.cancer-pku.cn/#index). The expression of POU6F2-AS1 in cell lines of different tissue origins were analyzed based on the Cancer Cell Line Encyclopedia (CCLE) database (<https://sites.broadinstitute.org/ccle/>). The OS survival curves of POU6F2-AS1 in GSE16125 database was conducted in lncCAR website (https://lncar.renlab.org/explorer). Lipid metabolism-related genes (LMGs) were collected from the “metabolism of lipids” in Reactome database (<https://reactome.org/download-data/>). JASPAR (<http://jaspar.genereg.net/>) was used to predict potential transcriptional binding sites for YBX1 in the FASN promoter region. RBPDB (<http://rbpdb.ccbr.utoronto.ca/>) and RBPmap (<http://rbpmap.technion.ac.il/>)were used to predict potential RBPs binding with POU6F2-AS1. catRAPID (<http://s.tartaglialab.com/page/catrapid_group>) was used to predict the binding region of POU6F2-AS1 to YBX1. SRAMP (<http://www.cuilab.cn/sramp>) was used to predict the m^6^A modification site of POU6F2-AS1.

**Application of palmitic acid (PA) and orlistat**

Commercially synthesised PA (10mM in DMSO) and FA synthetase inhibitor orlistat (10mM in DMSO) were purchased from GLPBIO (CA, USA). A working solution of 50 μM PA or orlistat was prepared according to the manufacturer’s instructions and used to assess their effect on the proliferative capacity of CRC cells.

**Liquid chromatography mass spectrometry (LC-MS)-based FA analysis**

HCT116 cells (1×10^7^) bearing control or POU6F2-AS1 knockdown were washed with PBS and collected into centrifuge tubes, completely aspirated out of PBS, snap-frozen in liquid nitrogen and used for lipid extraction. Centrifuge tubes containing cell precipitates were placed on dry ice and add 400 μL metabolite extraction solution (acetonitrile/methanol/water (2/2/1, v/v/v)) containing 0.25 μg/mL FA16:0-d3 to the tube and then sonicate for 5 min. Take 200 μL supernatant and then add 800 μL methyl tert-butyl ether, vortex 60s, centrifuge 15 min at 14000 rpm at 4 °C. Pipette the supernatant and vaccum-dried and dissolved with 120 μL of ACN/isopropanol/water (v/v/v=65:30:5) for LC-MS. The fatty acids were then quantified using Sciex TripleTOF 6600 (SCIEX, MA, USA) equipped with a Shimadzu LC.A BEH C18 (100×2.1mm, 1.7µm, Waters Co., MA, USA). 10 μL of dissolved sample was loaded into the column and the eluted by binary mobile phase A (acetonitrile/water solution, v/v=6:4, containing 10mM ammonium acetate) and B (isopropanol/acetonitrile solution, v/v=9:1, containing 10mM ammonium acetate) with flow rate as 0.30 mL/min. The elution gradient was conducted as follows: initial 40% B maintained for 1.5min, linearly increased to 85% B from 1.5 to 10.5min, maintained at 85% B for 3.5 min, then to 100% B from 14.0 to 14.1 min, maintained at 100% B from 114.1 to 15.0 min, and then decreased to 40% B from 15.0 to 15.2 min, finally maintained at 40% B from 15.0 to 18min. MS parameters were as follows: the declustering potential was set to -80 V; collision energy was set to -10 V; and ion spray voltage was set to -4500 V , with a mass range from m/z 50 to 1000. The pressure of ion source gas 1, ion source gas 2, and curtain gas was set to 50, 50, and 35 psi, respectively, and an interface heater temperature was at 500°C. The chromatographic and mass spectrometry data were acquired by Analyst TF 1.8.1 software (SCIEX, MA, USA), in ESI-modes with information-dependent acquisition (IDA). The raw data were processed using the open-source software MS-DIAL (v.4.9; RIKEN Center for Sustainable Resource Science, Saitama Prefecture, Japan). Finally, the data were normalised according to the internal standard and protein concentration.
